# Anthropic Activity Markers 2.0: A Shift Towards Compositional Data Analysis

**DOI:** 10.1007/s10816-026-09799-9

**Published:** 2026-06-10

**Authors:** Abel Ruiz-Giralt, Stefano Biagetti, Carla Lancelotti, Óscar Parque, Antonios Koutroumpas, Keelie S. Rix, Jordi Ibáñez-Insa, Marco Madella

**Affiliations:** 1 Culture, Archaeology and Socio-Ecological Dynamics Research Group, Universitat Pompeu Fabra, C. Ramon Trias Fargas 25-27, 08005 Barcelona, Spain; 2 ICREA, Passeig Lluís Companys 23, Barcelona, Spain; 3 School of Geography, Archaeology and Environmental Studies (GAES), University of the Witwatersrand, 1 Jan Smuts Avenue, Braamfontein 2000, Johannesburg, South Africa; 4 Geosciences Barcelona (GEO3BCN-CSIC), Lluís Solé I Sabarís S/N, 08028 Barcelona, Spain

**Keywords:** Anthropic Activity Markers, Soil Geochemistry, Compositional Data Analysis, Geostatistics, Ethnoarchaeology

## Abstract

Anthropic Activity Markers (AAMs) were formalized over 10 years ago as a toolkit to infer human activities from biological and geochemical signatures preserved in sediments. This paper presents AAMs 2.0, a revised analytical framework that integrates compositional data analysis (CoDA) and geostatistics, as the appropriate statistical foundations for this approach. We first outline the theoretical motivation of this framework, demonstrating that treating geochemical concentrations as absolute values generates statistical artefacts that limit analytical reliability. Using the ethnoarchaeological dataset of Rondelli *et al*. (2014) as a test case, we illustrate how CoDA principles can be applied to revisit established interpretations. With this approach, we can identify meaningful compositional relationships without relying exclusively on heuristic element combinations, yielding results that are more statistically robust. Ultimately, AAMs 2.0 reframes activity markers as relational, ratio-based signals in space, providing a more rigorous foundation for archaeological inference.

**Supplementary Information:**

The online version contains supplementary material available at https://doi.org/10.1007/s10816-026-09799-9.

## Introduction

About a decade ago, Rondelli *et al*. (2014) formalised the concept of *Anthropic Activity Markers* (AAMs) and established a framework for identifying human activities through their biological and geochemical traces preserved in archaeological sediments (*e.g*., chemical elements, plant micro-remains, or biomolecules). In this paper, we focus on geochemical AAMs. The potential of this approach lies in its capacity to reveal features and practices otherwise archaeologically invisible, because of taphonomic loss or the very nature of the practices themselves. Ultimately, the goal is to reconstruct human behaviour from archaeological deposits by using dependable signatures of human activity through an interpretative framework developed from experimental and ethnoarchaeological approaches. The AAMs framework is based on two key assumptions:

1. That specific activities repeated over time create unambiguous and identifiable proxy signatures, no matter how those activities are performed; and.
2. That these signatures are broadly consistent within and between sites, no matter the chronological, cultural or geographic context.

The foundations of this research approach were established in the late 1970s, when a series of ethnoarchaeological studies (*e.g*., Hodder & Orton, 1976; Halstead *et al*., 1978; Hassan, 1978; Kent, 1987; Kroll & Price, 1991) highlighted that people tend to consistently use specific areas of their living spaces and/or landscapes for particular activities. These early works demonstrated that such spatial patterning could be archaeologically detected through the distribution of macroscopic remains, especially lithics and faunal material (Lancelotti *et al*., 2017). In parallel, the use of soil geochemistry as a tool to detect activity areas demonstrated that a repetitive use of space for certain activities leaves identifiable geochemical signatures in the sediments that are representative of such practices (Barba, 1978, 1986, 2007; cited in Lancelotti *et al*., 2017). Geochemical analysis of archaeological sediments can provide reliable evidence about past human behaviour, especially when contextualised through experimental and ethnoarchaeological frameworks (Pecci *et al*., 2013, 2017; Rondelli *et al*., 2014; Zurro *et al*., 2017; Gur-Arieh *et al*., 2019; Sulas *et al*., 2019; Biagetti *et al*., 2021; see also Lancelotti *et al*., 2017, and references therein).

In the last 10 years, the AAMs framework has evolved into a structured, inferential system built from multiple proxies, explicitly linking microscale signatures to specific human activities (*e.g*., Rondelli *et al*., 2014; Zurro *et al*., 2017; Pecci *et al*., 2018; del Puerto *et al*., 2022; Seitsonen & Égüez, 2021). Among the available methodologies, soil geochemistry has gained significant popularity in the last few years, mostly due to the rapid expansion and increasing accessibility of advanced multi-elemental analytical techniques (see Rouhani *et al*., 2024). In practice, geochemical AAMs have often been analysed through single-element distributions, element combinations, correlation analysis, principal component analysis, and spatial interpolation, generally treating elemental concentrations as independent variables. This paper revisits that analytical logic by comparing the results obtained from such non-compositional approaches with those produced through a workflow based on compositional data analysis (see Martín-Fernández *et al*., 2015a; Greenacre & Wood, 2024). In doing so, we use the Rondelli *et al*. (2014) dataset to show how different statistical assumptions lead to different archaeological outcomes. This comparison demonstrates that treating elemental data as relational rather than absolute does not simply modify the analytical procedure, but changes what can be identified as a geochemical activity marker.

## State of the Art

### Geochemical Approaches to the Study of Anthropogenic Sediments

Geochemical analysis of ancient and contemporary anthropogenic sediments for archaeological purposes is a long-established practice, pioneered by O. Arrhenius in the 1920–1930s. His work on phosphate analysis in a large-scale geological survey laid the foundation for the use of geochemical residues in site identification (Arrhenius, 1929). Building on the idea that human activities alter soil properties in ways that can be chemically identified, new investigations expanded the range of elements analysed in order to overcome the limitations of relying solely on phosphates (*e.g*., Provan, 1973; Proudfoot, 1976; Shackley, 1975; Eidt, 1977; Mejia Perez & Barba, 1988; Deotare & Kshirsagar, 1993; Deotare *et al*., 1999; Holliday & Gartner, 2007). More recently, such an approach became focused on multi-elemental characterization of anthropogenic sediments. The complexity of the processes shaping geochemical signatures has led to the development of experimental and ethnoarchaeological studies conducted in controlled or semi-controlled settings (see Lancelotti *et al*., 2017 and references therein), such as present-day houses and settlements, where the relationships between human activities and their geochemical expressions can be more directly observed and evaluated, making it possible to identify which elements are consistently associated with specific anthropic activities. This approach has now become widespread in archaeological and ethnoarchaeological investigations for both site prospection and the study of geochemical variation between the different areas of a site (*e.g*., Middleton & Price, 1996; Entwistle & Abrahams, 1997; Entwistle *et al*., 1998, 2000; Middleton, 2004; Oonk *et al*., 2009a, 2009b; Wilson *et al*., 2005, 2008, 2009; Misarti *et al*., 2011; Davis *et al*., 2012; Dirix *et al*., 2013; Gauss *et al*., 2013; Rondelli *et al*., 2014; Lubos *et al*., 2016; McKinley *et al*., 2016; Zurro *et al*., 2017; Horák *et al*., 2018, 2023; Ginau *et al*., 2020; Janovský *et al*., 2020, 2022; Salisbury, 2020; Save *et al*., 2020; del Puerto *et al*., 2022; Biagetti *et al*., 2021; Danielisová *et al*., 2022; Holmqvist & Ilves, 2022; Kalkan *et al*., 2024). The growing use of analytical techniques that enable simultaneous multi-element analyses, such as X-ray fluorescence (XRF) and inductively coupled plasma (ICP)-based techniques (ICP-AES, ICP-OES, ICP-MS), has strengthened this approach. These techniques produce comprehensive datasets that allow for the creation of multi-element maps and core scans, which can be used to identify spatial patterns both horizontally across sites and vertically through stratigraphy. Furthermore, investigations now implement multivariate statistical analyses to assist in disentangling the complex web of natural and anthropogenic geochemical signals of sediments (see Bintliff & Degryse, 2022; Rouhani *et al*., 2024, for recent reviews). These developments have significantly expanded the analytical potential of geochemical approaches in archaeology. However, they also raise important questions regarding the interpretation of increasingly complex datasets.

### Limitations of Geochemical Analysis in Anthropogenic Sediments

Despite these methodological advances, significant interpretative challenges persist. One of the most persistent and widely debated issues in soil geochemistry is the problem of equifinality (see Lyman, 2004; Pecci *et al*., 2017). Elemental concentrations observed in anthropogenic contexts can arise from a diverse number of post-depositional processes and the geological properties of the soil (Bintliff & Degryse, 2022). This is critical for the present study: while early works established direct links between intensity of chemical residues and specific activities (*e.g*., Middleton & Price, 1996), in practice the observable geochemical patterns are always expressed within specific sedimentary contexts and hence are not fixed (see Oonk *et al*., 2009a, 2009b, 2009c; Lancelotti *et al*., 2017; Bintliff & Degryse, 2022). As a result, for nearly every element (or group of elements) that have been identified as a marker of a specific activity—*e.g*., phosphorus for organic waste, metals for craft production—alternative explanations have also been suggested (*e.g*., Middleton, 2004; Oonk *et al*., 2009a; Lubos *et al*., 2016; Pastor *et al*., 2016; Save *et al*., 2020; see Bintliff & Degryse, 2022 for a recent review). Additionally, these signatures must be understood as temporally dynamic. As noted by Bintliff and Degryse (2022), growing empirical evidence challenges the assumption that recurrent formation processes directly produce consistent markers. Even if similar activities introduce comparable signatures into the sediment, their geochemical expression can change rapidly once incorporated into a specific soil matrix, as it is mediated by background composition, sediment properties, moisture, pH, biological activity, cleaning practices, and post-depositional processes. Consequently, similarities across sites are not expected to appear as identical elemental combinations, concentrations, or thresholds. Rather, they are more likely to emerge as recurring relational structures between components, shaped by similar anthropic activities but expressed under different local conditions.

Anthropogenic soils geochemistry must also contend with the spatial structure of the data. Human activities rarely occur in isolation, but they are embedded in spatial contexts that shape (and are shaped by) them. Studying the spatial distribution of chemical elements through geostatistical methods has significantly advanced the potential of the AAMs framework: by applying techniques such as variogram analysis and kriging, researchers have been able to model spatial autocorrelation and interpolate between samples (*e.g*., Rondelli *et al*., 2014; Negre *et al*., 2016; Biagetti *et al*., 2021, 2026; see Lloyd & Atkinson, 2004, 2020 for a recent review), thereby achieving more nuanced insights in the social organisation of space by past human communities.

Perhaps most critically, the compositional nature of multi-elemental geochemical datasets has often been overlooked, with only a few exceptions (e.g., Horák et al., 2018, 2023; Ginau et al., 2020; Biagetti et al., 2021, 2026; Danielisová et al., 2022; Janovský et al., 2022). The theoretical and methodological approach often adopted in anthropogenic soil geochemistry has so far placed undue emphasis on the direct correlation between specific activities and absolute concentrations, whether considered individually or across multiple elements. This perspective treats elemental concentrations as independent variables, ignoring the inherently compositional nature of geochemical data. The concentrations of elements in a sample are not independent, but they exist in a constant-sum constraint, where all variables sum to a unit value (1, 100%, or 1,000,000 ppm) (see Pingitore & Engle, 2022). In other words, single element measurements carry relative information: if the proportion of one component increases, the proportion of at least one other component must decrease to maintain the constant sum, regardless of whether true correlation between the two exists. Traditional statistical methods are fundamentally incompatible with this data structure because they assume data can vary independently and freely across an unbounded range, from negative to positive infinity. As a consequence, the interpretability and validity of individual components have been repeatedly questioned, owing to their susceptibility to create a range of statistical artifacts. According to McKinley *et al*. (2016), these include spurious correlations (*i.e.* apparent statistical relationships between variables that arise not from a genuine correlation but from mathematical constraints); interpretative dependence on other, often unreported components; and sensitivity to unit choices (see also Pearson, 1897; Chayes, 1960; Aitchison, 1982, 1986; Egozcue *et al*., 2003).

Importantly, the challenges associated with the analysis of compositional datasets are not unique to anthropogenic soils geochemistry. Similar issues related to closed arrays and constant-sum constraints have long been recognised across multiple archaeological subfields, including zooarchaeology (*e.g*., Grayson, 1984; Lyman, 2008), ceramic analysis (*e.g*., Orton & Hughes, 2013) and, most importantly, archaeometry (*e.g*., Tangri & Wright, 1993; Aitchison *et al*., 2002; Baxter *et al*., 2005; Baxter & Freestone, 2006; Baxter, 2008, 2015; Buxeda i Garrigós, 2008), where the compositional nature of multi-elemental datasets has been widely discussed. Across these areas, it is well established that changes in one component necessarily affect all others and cannot be uniquely interpreted, generating interpretive ambiguities if not properly addressed. To address this, archaeologists have focused not only on adopting appropriate statistical tools, but also on the selection of meaningful ratios to operationalise specific hypotheses about past human behaviour: in zooarchaeology, for instance, ratios such as wild to domestic taxa, cattle to sheep proportions or contrasts between skeletal element groups are used to explore subsistence strategies, herd management and butchery practices (Lyman, 1994, 2008). Similarly, research in material sourcing routinely uses chemical ratios such as Ca/Sr or Zr/Sr to distinguish clay and obsidian sources respectively (*e.g*., Hughes & Smith, 1993; Wilson, 1978), but also the provenance of metal objects (Pernicka, 2014). These theory-based approaches, however, define the analytical space a priori, potentially limiting the exploration of alternative relationships (see Dubova *et al*., 2026 for a recent discussion). This limitation becomes particularly relevant in increasingly complex, high-dimensional datasets such as multi-elemental compositional data, which can benefit from applying data-driven approaches instead (see Hein & Kilikoglou, 2020; Hein, 2026; for a recent example of the use of data-driven methods in provenance studies of archaeological materials).

Despite the substantial body of work in other subfields, the implications of compositional data have only recently begun to be explicitly acknowledged within AAM geochemistry (see Ginau *et al*., 2020; Biagetti *et al*., 2021; Danielisová *et al*., 2022; Holmqvist & Ilves, 2022; Janovský *et al*., 2022; Horák *et al*., 2023). Earlier studies often identified anomalous patterns related to the compositional nature of multi-elemental data. For instance, Middleton and Price (1996) described unexpected inverse relationships between Ti and the rest of the chemical elements from house floors from Oaxaca (Mexico), interpreting them as statistical outliers resulting from successive construction events rather than a product derived from the compositionality of the data. Lancelotti and Madella (2012) observed systematically higher concentrations of elements in ashed dung compared to fresh dung, which they correctly associated with the loss of organic matter. The additional unexplained variation they reported can, however, be similarly understood as spurious correlation arising from the compositional structure of the data. Finally, Rondelli *et al*. (2014) recorded significantly high correlations between elements in the house floors of an ethnographic site from northern Gujarat (India), leading the authors to suggest that it was not possible to identify significant chemical combinations associated with human activity due to the effects of the specific preparation of the floor plaster. All these results, however, are largely the byproduct of the compositional nature of the data: they do not show actual patterns in elemental content, but rather they are statistical artifacts produced by the closure effect (*i.e*. the distortion that emerges in compositional data due to constant-sum constraints, which alters covariance relationships so that the total covariance of a given component with all the others is equal to the negative of its own variance). This constraint creates dependencies among variables, violating the assumption of independence required by standard statistical methods and leading to biased covariances and spurious correlations in raw compositional datasets (see Filzmoser *et al*., 2018, and references therein).

Taken together, these challenges call for rethinking how soil geochemical data are conceptualised within AAMs research: rather than treating elemental concentrations as absolute values, they must be analysed as inherently relative. In geochemistry, this has long been addressed through the use of ratios and log-ratio expressions as fundamental units of analysis (*e.g*., Urey, 1947; Nesbitt & Young, 1989; Schedl, 1998). This, in turn, requires multivariate, statistically robust analytical approaches that acknowledge the compositional nature of the data while integrating their spatial dimension.

### Compositional Data Analysis Framework

**Fig. 1:**
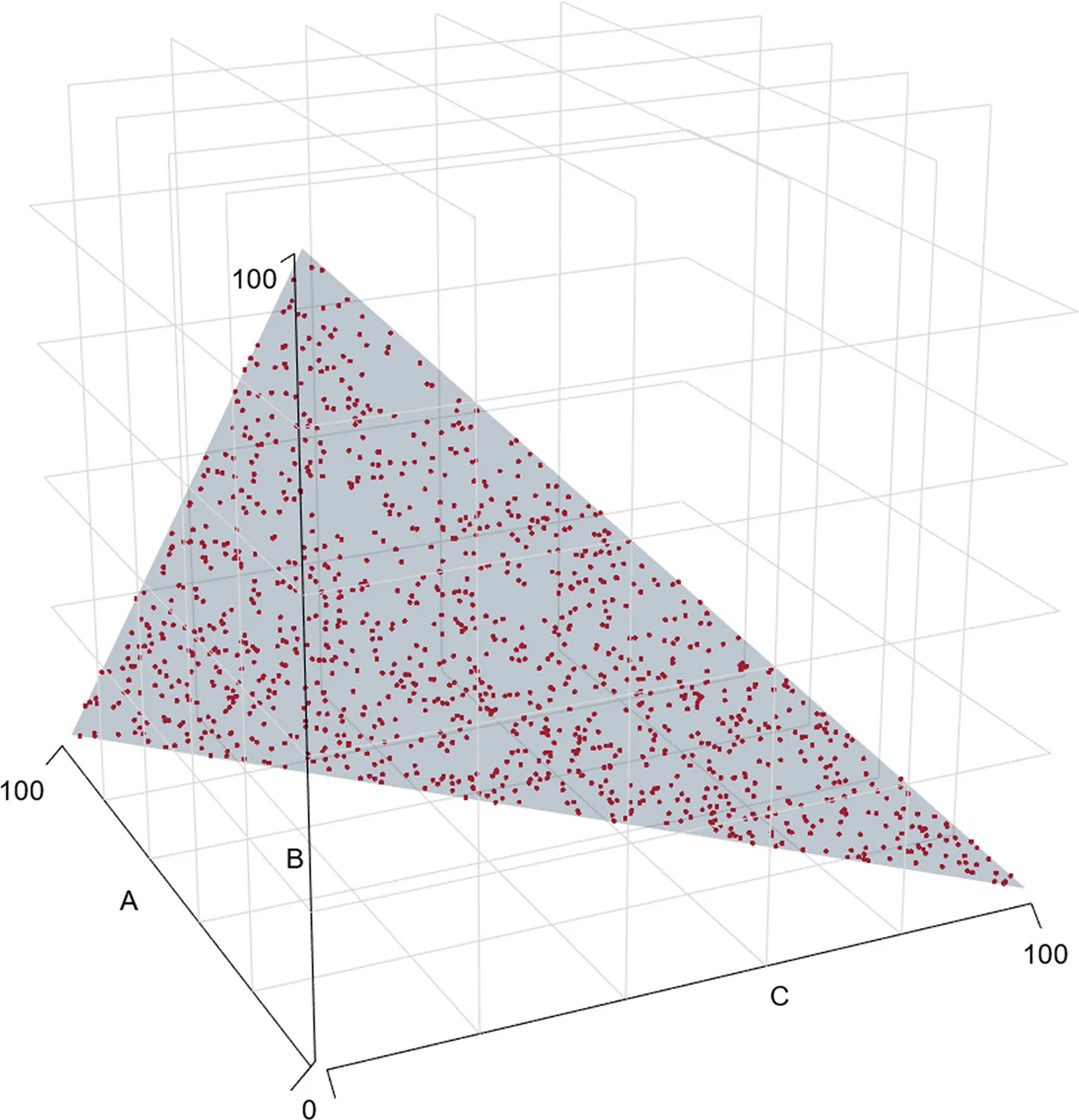
3D representation of a random dataset of three-part compositions (**A**, **B**, **C**). Due to the closure effect, all valid compositions (red dots) are confined to a two-dimensional space called the simplex, which appears as a closed triangular surface embedded within the three-dimensional Euclidean space. For two-part compositions, the simplex reduces to a line; while for four-part compositions it is a tetrahedron. Compositions with five or more parts extend their simplex into high-dimensional spaces that cannot be fully visualized

To address these limitations, a shift towards analytical frameworks that explicitly account for the compositional nature of geochemical data is required. Compositional Data Analysis (CoDA) provides a mathematically coherent framework for handling data constrained by constant-sum and relative scale properties (see Aitchison, 1982, 1986; Egozcue *et al*., 2003; Van Den Boogaart & Tolosana-Delgado, 2013; Greenacre, 2018; Filzmoser *et al*., 2018; Tolosana-Delgado & Mueller, 2021). In doing so, CoDA enables the systematic exploration of relationships between components in high-dimensional datasets, complementing theory-driven approaches. The CoDA paradigm (Aitchison, 1982) includes a rigorous mathematical framework for the analytical practices that were being used in fields such as geochemistry since the 1940–1950s (*e.g*., Urey, 1947). Aitchison (1982) demonstrated that this data does not reside in ordinary Euclidean space, where variables can vary freely and independently, but in a specific sample space known as *the simplex*, a closed geometric space which inherently accounts for the constant sum and positivity constraints (Fig. 1, see also Supplementary Materials 1 (SM1) for an extended glossary of key concepts). This geometry also reduces the dimensionality of the data: a composition with *D* parts has only *D*–1 degrees of freedom, since the value of any part is fixed once the others are known, reflecting the relational rather than absolute nature of the data (Aitchison, 1986, 1999; see also Filzmoser *et al*., 2018; Greenacre *et al*., 2023). For instance, a three-part composition can be fully expressed in two dimensions, which is why it is naturally represented in a ternary diagram.

This change in geometry leads to a fundamental shift in interpretation. In Euclidean space, distances between points capture absolute differences, and variables can vary independently. In the simplex, however, the parts are interdependent, and the meaningful information lies in their ratios. This implies that two samples with different elemental concentrations may in fact represent the same composition if their internal proportions are identical. In compositional terms, they are part of the same *equivalence class* of compositions: a set of data points that, although expressed in different units or scaled to different totals, carry exactly the same relative information (see Barceló-Vidal and Martín-Fernández, 2016). Recognising this property, ratios, rather than absolute values, are the fundamental carriers of information in compositional data. Subsequent work has built on this foundation by formalising two key principles that any analysis of compositional data must respect: scale invariance and subcompositional coherence (Filzmoser *et al*., 2018; see SM1 for details).

- *Scale invariance*: a composition is defined only by the ratios between its parts, not by its absolute magnitude. Analyses must therefore yield the same results if all parts are multiplied by the same positive constant.
- *Subcompositional coherence:* the information contained in any subset of components is consistent with that of the full composition. Analyses must therefore give the same interpretation of a subset whether it is considered on its own or within the entire composition.

These principles ensure that data analysis focuses on the meaningful relational structure of the data itself, avoiding distortions introduced by treating compositions as if they were unconstrained variables. Beyond this, the simplex has a rich algebraic–geometric structure: Aitchison geometry defines operations such as *perturbation*, which is analogous to vector addition, and *powering*, which scales compositions—both of which preserve the compositional nature of the data while enabling formal statistical operations (Egozcue & Pawlowsky-Glahn, 2006; see SM1 for details). Additional core concepts include the *centre* of a composition (the closed geometric mean, serving as the analogue of an “average composition”) and *variation matrices* (capturing dispersion through the variances of all pairwise log-ratios, and forming the basis for measures such as *total variance*), which are essential to describe central tendency and dispersion in a way that is consistent with the simplex geometry (Pawlowsky-Glahn & Egozcue, 2002; Filzmoser *et al*., 2018; see SM1 for details). Together with these operations, Aitchison geometry also defines its own way of measuring similarity and difference: a specific distance metric (the *Aitchison distance*), and associated notions of length (*norm*) and angle (*inner product*) (Aitchison, 1992; Filzmoser *et al*., 2018; see SM1 for details). Altogether, these tools provide the mathematical backbone for CoDA, ensuring that statistical analyses are consistent with the geometry of the simplex.

While this framework defines the internal logic of the simplex, scientific practice, in archaeology as in most fields, often relies on Euclidean-based standard multivariate statistics as the main analytical toolkit. To make these methods applicable to compositional data, a bridge is required. This is provided by log-ratio transformations, which translate the relative information contained in a composition into values in Euclidean space (Martín-Fernández *et al*., 2015a). By converting ratios into logarithms, these transformations eliminate the closure effect and make it possible to apply conventional statistical methods without violating the structure of the data. In this way, key concepts of Aitchison geometry take on familiar statistical forms: for example, the centre of a composition corresponds to the mean of the log-ratio coordinates, and the Aitchison distance translates into the Euclidean distance. Different transformations are available (Aitchison, 1982; Egozcue *et al*., 2003; see SM1 for details):

- *Additive log-ratio* (alr): expresses each part relative to a chosen reference. This can be useful for targeted comparisons, particularly when a meaningful denominator exists (*e.g*., expressing element concentrations relative to the unmeasured fraction), though results remain sensitive to the choice of denominator.
- *Centered log-ratio* (clr): uses the geometric mean as a reference, providing an intuitive picture of the whole composition. It is well suited for exploratory analyses and visualisation, but its lack of orthogonality limits formal modelling.
- *Orthonormal log-ratio* (olr), previously known as isometric log-ratio (ilr): map compositions into orthonormal coordinates, ensuring statistical coherence and preserving distances in the Aitchison geometry. In practice, olr coordinates are derived from *log-ratio balances*—that is, linear combinations of log-transformed parts with coefficients summing to zero and scaled to form an orthonormal basis in the simplex (Martínez-Fernández *et al*., 2018; see SM1 for details). Each log-ratio balance quantifies the relative dominance between two groups of components, providing interpretable contrasts within the composition. While less intuitive to visualise directly, olr coordinates yield mutually uncorrelated (orthogonal) variables that are well suited for regression, classification, and other multivariate techniques.
- *Symmetric log-ratio balances*: introduced by Kynčlová *et al*. (2017), this approach defines a specific case of orthonormal log-ratio coordinates that treat any pair of components symmetrically with respect to all others. Each balance expresses one part relative to the geometric mean of the remaining parts, capturing how a component dominates (or is dominated by) the rest of the composition. This symmetry avoids the directional bias of sequential binary partitions while preserving orthogonality in the Aitchison geometry, making it well suited for correlation analysis between compositional parts (see also Filzmoser *et al*., 2018: 52–56).

Although these transformations differ in formulation, they share the same foundation: representing compositional data in a way that makes conventional statistical methods applicable without violating the constraints of the simplex. This process can be reduced to three practical steps (see Mateu-Figueras *et al*., 2011 for details):

i) Represent compositional datasets in the appropriate log-ratio coordinates.
ii) Apply Euclidean-based statistical analysis to the log-ratio coordinates as variables.
iii) Interpret the results both in coordinate space and in terms of the original compositional units after back-transforming the results.

In the following section, we operationalise these principles through a structured analytical workflow, illustrating how compositional and geostatistical methods can be applied to multielement geochemistry datasets in practice.

## From Theory to Practice

In this paper, we illustrate how a compositional approach fundamentally reshapes both the identification and spatial modelling of anthropic activity markers compared to traditional methods using the data published by Rondelli *et al*. (2014) as a test case. We follow the same analytical structure in order to evaluate the differences in results between a non-compositional and a compositional approach. Specifically, we contrast the results of the original paper against the application of a CoDA-based workflow for geostatistical analysis developed in the framework of the project *(Re)Constructing the Archaeology of Mobile Pastoralism* (CAMP) (see Biagetti *et al*. 2026 for further details). This workflow is implemented in R 4.3.3 (R Core Team 2024), using a set of packages for compositional data analysis and geostatistics (see Supplementary Materials 2, 3, 4 for a detailed list). Our aim is twofold: (i) to show how to implement a compositionally coherent data-driven approach that identifies elemental ratios to characterise the chemical signature of anthropic activity areas (instead of ad hoc ratio selection); and (ii), to demonstrate the integration of compositionally coherent geostatistical modelling, ensuring that spatial predictions remain consistent with the geometry of the simplex.

**Fig. 2:**
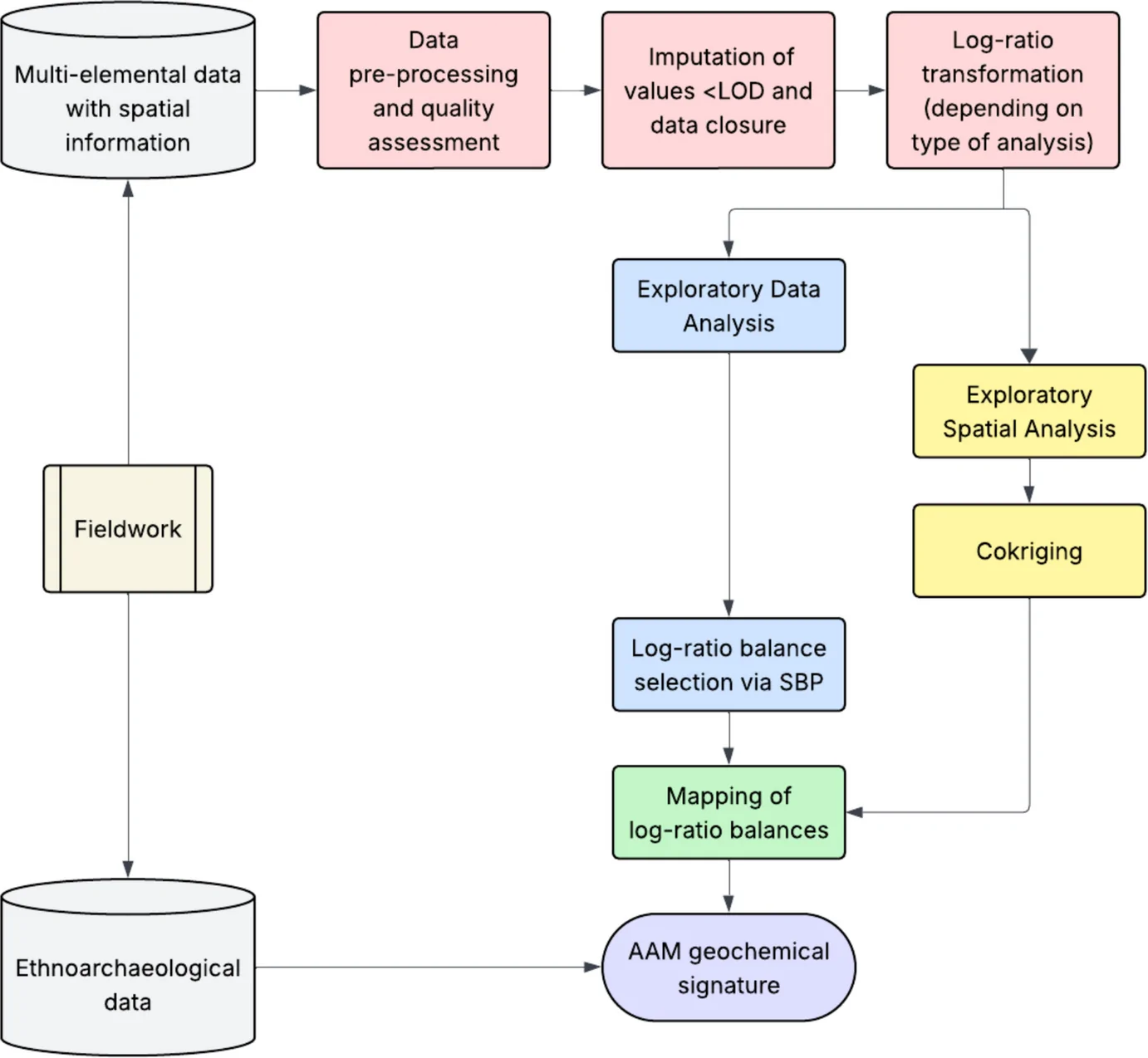
Workflow for the compositional and geostatistical analysis of multi-element pXRF data within the CAMP project, adapted to the current study

In general terms, the applied workflow follows a structured sequence that integrates multivariate compositional data analysis with geostatistics (Fig. 2). First, multi-elemental data are pre-processed by removing values below detection limits or with high uncertainty and introducing a residual component to ensure data closure. The dataset is then transformed to log-ratio coordinates and exploratory data analyses are carried out, including compositional correlation analysis and principal component analysis, in order to identify coherent patterns of elemental covariation and define meaningful geochemical associations. Group comparisons are performed through perMANOVA on Aitchison distances. Next, spatial analysis proceeds in two stages: an initial exploratory phase using interpolative methods to visualise general trends, followed by formal geostatistical modelling based on kriging techniques (specifically, cokriging). Finally, a Sequential Binary Partition (SBP) is used to select log-ratio balances via experimentally constructed dendrograms and mapped through cokriging. Results are interpreted against ethnoarchaeological observations to identify valuable geochemical balances that can serve as AAMs. Together, these steps provide a reproducible framework for analysing multi-element datasets while avoiding the distortions inherent to non-compositional approaches. A detailed account of the results is provided in Supplementary Materials 2, 3, 4.

The workflow presented here should be understood as the initial stage within a broader inferential process rather than as a self-contained hypothesis-testing framework. In contrast to approaches where analytical variables are defined a priori from specific behavioural expectations, our objective is to characterise the internal structure of the compositional system and identify statistically coherent contrasts (log-ratio balances) that capture its dominant patterns of variation. This step is particularly necessary in high-dimensional geochemical datasets where the relevant relationships between components are not yet well understood and where reference models remain limited. To do so, our approach is embedded within an ethnoarchaeological framework that provides independent observations of activity organisation, formation processes, and spatial practices, hence enabling an inductive approach to AAM analysis. These contextual data act as an external referential structure against which the identified compositional patterns can be evaluated, allowing the analytical results to be grounded in observed behavioural processes rather than treated as purely data-driven artefacts. Once these compositional structures have been identified and contextualised, they can be formalised into more explicit, theory-driven contrasts in future applications, particularly as reference datasets and background compositions become better characterised. In this sense, the approach proposed here is not opposed to hypothesis testing, but rather precedes it: it provides a systematic way to define meaningful analytical variables that can later be used to operationalise and test archaeological hypotheses across contexts.

### Case Study: Jandhala, Gujarat

**Fig. 3:**
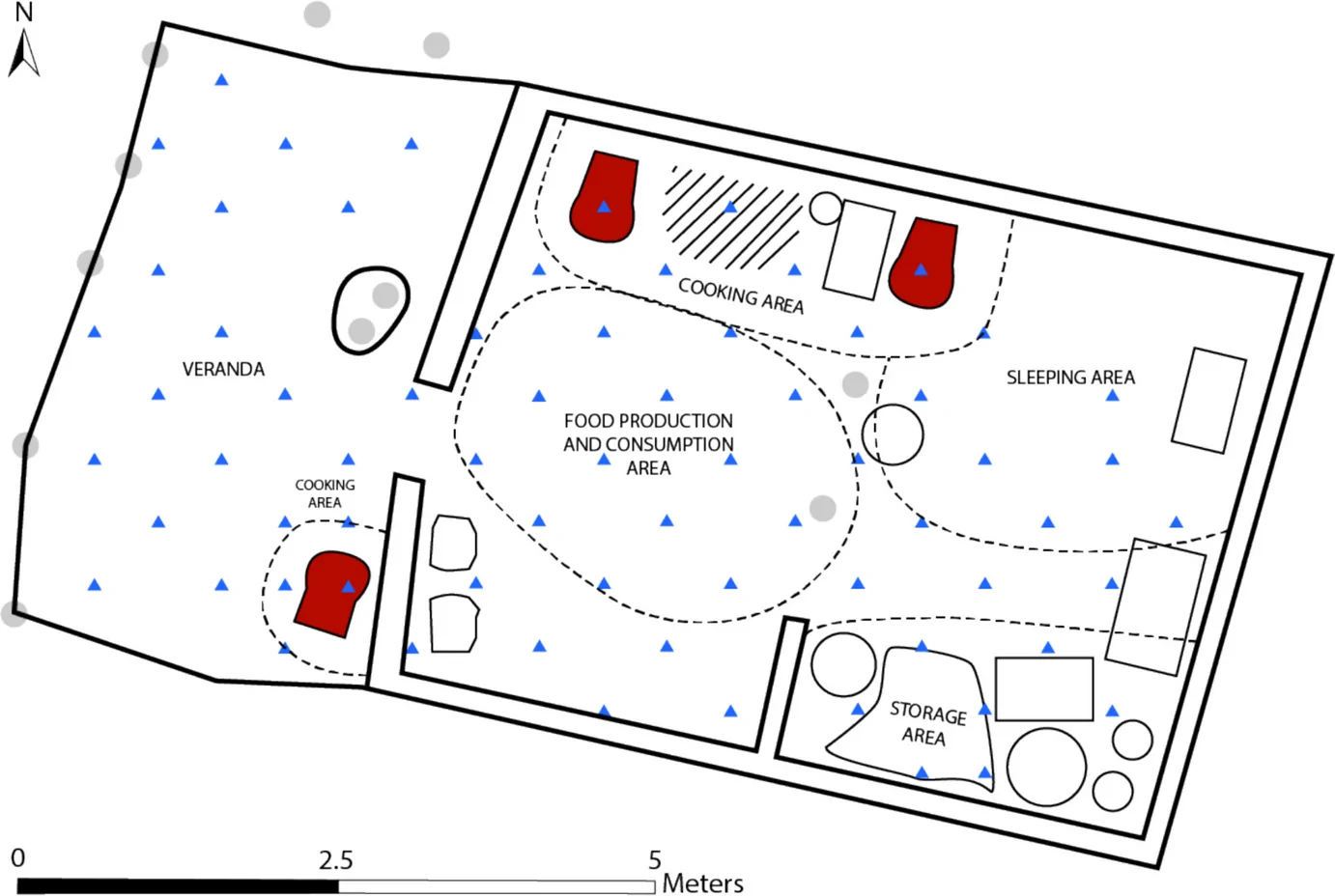
Detail of House A in Jandhala, Gujarat, India. The map shows the sample location and domestic features (fireplaces in red) as observed during sampling and the main activity areas as observed by the team and explained by the family living in the house (see Rondelli *et al*., 2014: 485 for details)

Rondelli *et al*. (2014), as part of the NoGAP project, focused on the analysis of a domestic structure, *House A*, within a farmers’ compound in the village of Jandhala in the Patan district of North Gujarat, India. At the moment of sampling (2010), the building had been continuously inhabited since its construction after the 2001 earthquake. The building used traditional local materials and techniques (e.g., wattle-and-daub walls, floors plastered with clay, sand, dung and water) and it was maintained through regular re-plastering events. These practices, documented through unstructured interviews with household members and micromorphological analysis of floors, provided the context to understand how geochemical residues were produced (which activity) and became then part of the domestic surfaces during a decade of use. The house consisted of a semi-open veranda at the front and an internal area arranged as a single rectangular room with a partial division midway (see Rondelli *et al*., 2014: 484 for details). The NoGAP team recorded the topography of the house with a total station (Fig. 3).

Sampling followed a systematic grid strategy: 70 sediment samples were collected on a 1 m × 1 m grid (Fig. 3). Each sample was extracted with a 5-cm-diameter hollow metal core to a depth of 2 cm, ensuring the sampling of the full sequence of plastering episodes above the floor foundations. The samples were then homogenised by grinding to a fine powder with an agate mortar, quartered, and divided into two aliquots: one used for spot tests and the other for multi-element analysis with Inductively Coupled Plasma–Atomic Emission Spectrometry (ICP-AES). Spot tests targeted phosphates, fatty acids, and protein residues (see Rondelli *et al*., 2014: 486 for methodological details). No background or control samples (*e.g*., surrounding soils or construction materials) were collected in the original study. Here, we focus on the results of the ICP-AES, which were produced by ALS Laboratory Group (Seville, Spain) after aqua regia digestion of the samples to determine the concentrations of 35 major elements. 11 elements were excluded due to more than 90% of all measurements falling below the instrument’s detection limits, including Be, Cd, Ga, Hg, La, Mo, Sb, Th, Tl, U, and W. The analysed dataset is provided as Supplementary Information in the original publication, raw data available here as Supplementary Materials 4. In order to enable direct comparability with the original publication, we retained the same set of elements reported by Rondelli *et al*. (2014), even though some components (notably Ag, but also Bi) are potentially problematic from an analytical standpoint due to the number of measurements below the limit of detection. We therefore add a cautionary note: best practice in future case studies is to incorporate expert knowledge to assess suspect elements prior to modelling to avoid over‑interpreting artefactual signals.

### Revisiting the Case Study with CoDA

The analysis by Rondelli *et al*. (2014), including correlation and variance analysis, as well as spatial interpolation by Inverse Distance Weighting (IDW), was carried out on log10-transformed data to eliminate the scale difference between values. However, this transformation treats the data as if they were independent variables on a ratio scale, thus ignoring the closure constraint. It is important to note that a log10 transformation differs fundamentally from the log-ratio transformations used in CoDA: whereas the latter express each part relative to the others within the composition, a log10 transformation merely rescales absolute values without addressing their interdependence. Rondelli and colleagues’ approach produced inconclusive results, leading the authors to state that the multi-element ICP-AES analysis did not allow the identification of reliable AAMs (Rondelli *et al*., 2014: 487). In the following, we revisit their dataset to explore why this was the case and how a CoDA-based approach can overcome these limitations. Since Jandhala’s dataset does not include background or control samples, all compositional patterns identified in this study are relative to the internal structure of the sampled dataset. Consequently, the results presented document relative differences between activity areas, but do not allow us to quantify absolute anthropogenic enrichment or to definitively distinguish between activity-related signals and variability in substrate or construction materials.

**Fig. 4:**
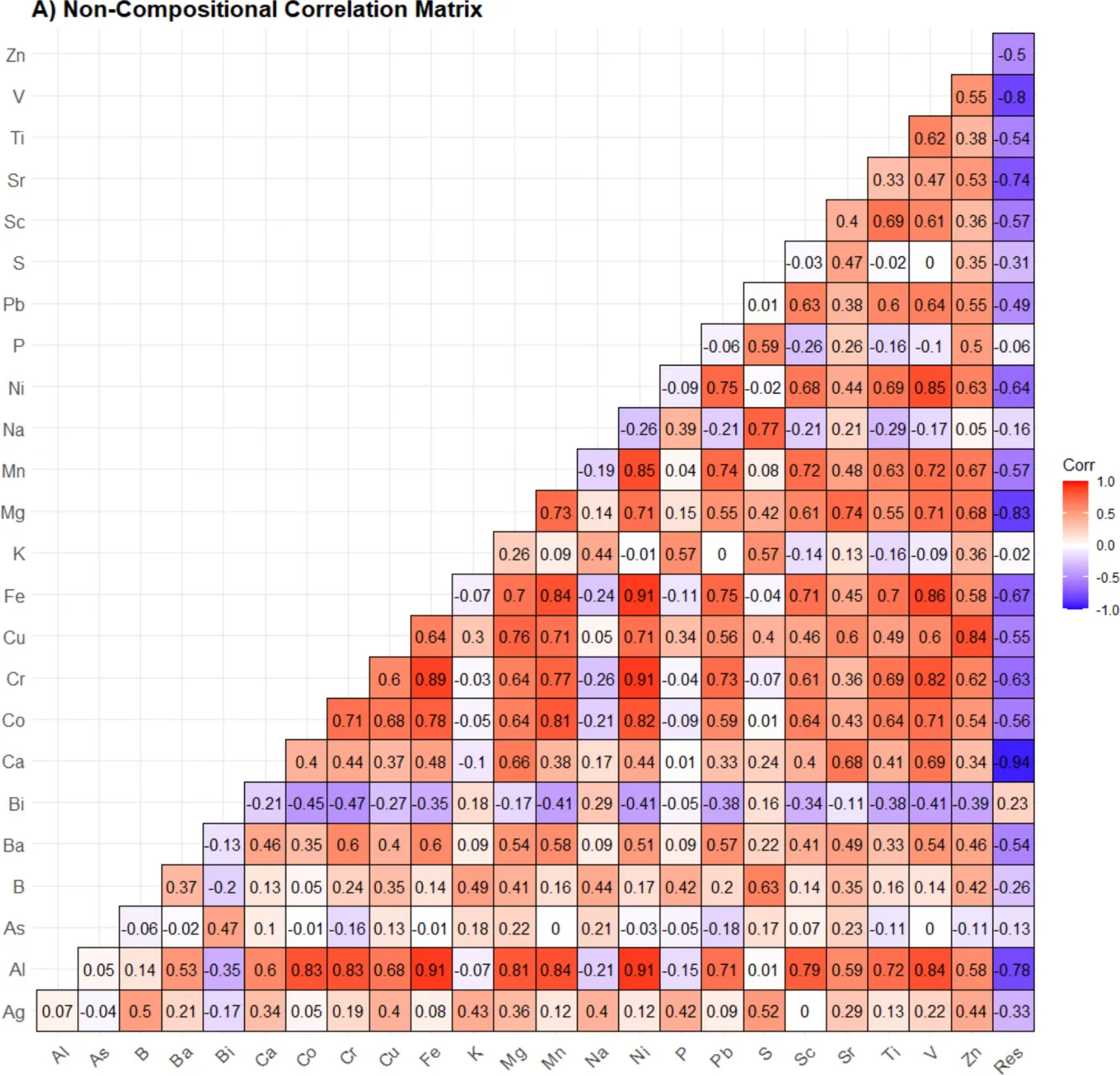

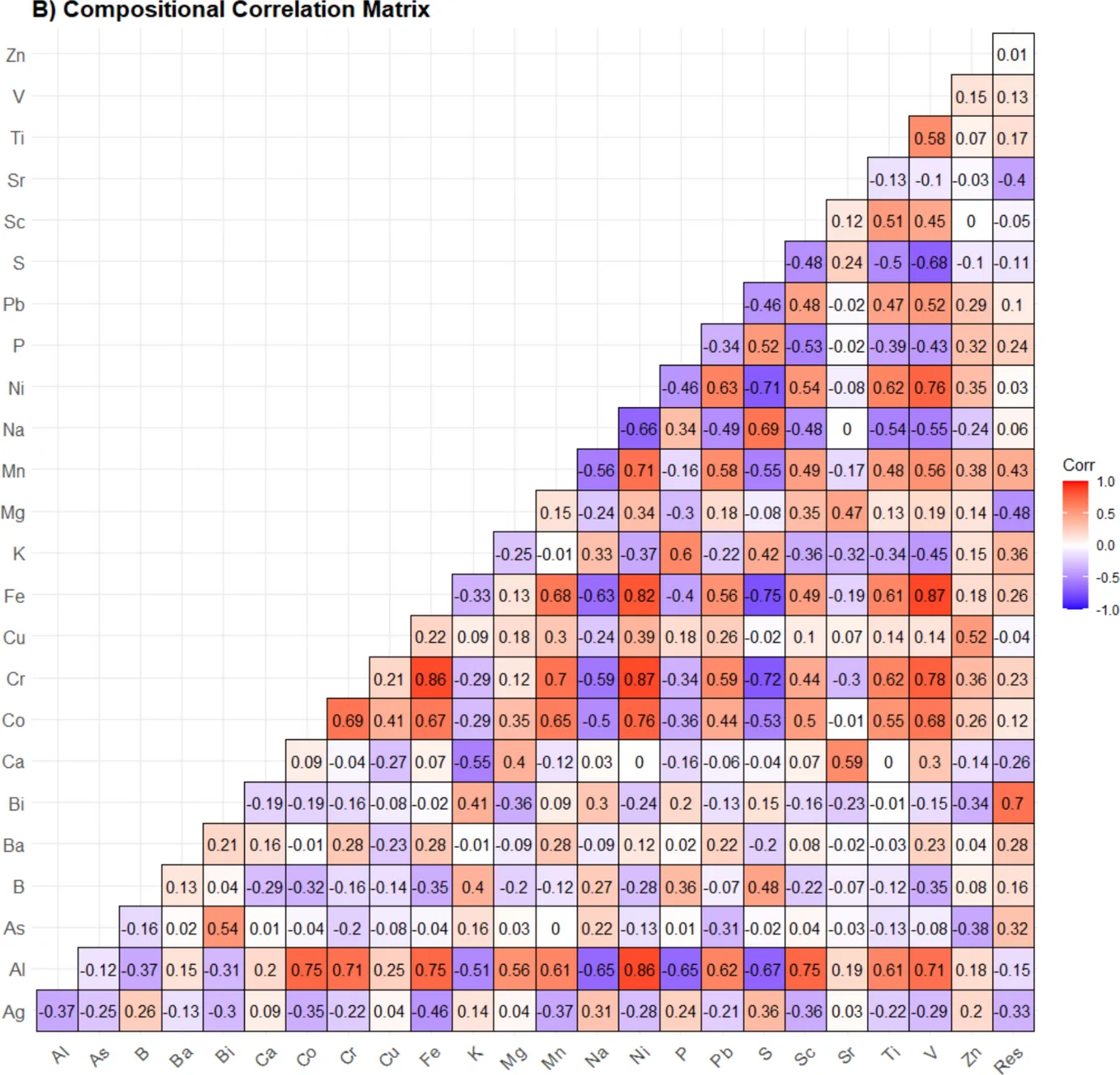
Correlation matrices employing log10 transformed values (**A**) vs. olr transformed values (**B**) of geochemical data from Jandhala (northern Gujarat), illustrating the effect of compositional transformation on correlation structure

***Correlation analysis*** by Rondelli *et al*. (2014) revealed strong positive correlations among nearly all measured elements, which prevented the identification of meaningful combinations of geochemical signatures (Fig. 4a). The authors considered this to be the result of the specific technique for preparing the plaster floor, which they argued generated uniformly high inter-element correlations. In reality, these correlations are largely spurious, as is typical for compositional data. To overcome the limitations of standard correlation analysis with compositional data, we applied the approach proposed by Kynčlová *et al*. (2017): in this method, symmetric log-ratio balances are used to construct orthonormal log-ratio coordinates, which express each part relative to the average of the remaining parts, hence capturing true relative associations and avoiding the distortions introduced by the constant-sum constraint (see Kynčlová *et al*., 2017; Filzmoser *et al*., 2018: 52–56 for detailed explanations). Once the dataset is expressed in log-ratio coordinates, the structure of covariation can be properly discerned (Fig. 4b). In this framework, positive correlations indicate that two components tend to co-dominate relative to the rest of the composition, whereas negative correlations indicate a trade-off in their relative contributions. Using a CoDA approach, we were able to distinguish a number of associated elements: for example, two main clusters of positively correlated elements were identified, *i.e*., Al-Co-Cr-Fe-Mn-Ni-Pb-Sc-Ti-V and B-K-Na-P-S. Strong negative correlations are also evident between these clusters; for instance, Al is negatively correlated with the B-K-Na-P-S cluster, while S shows negative correlations with the Al-Co-Cr-Fe-Mn-Ni-Pb-Sc-Ti-V group. Additional positive correlations were observed between Ca-Sr, As-Bi, and Cu-Zn. Importantly, these are correlations between log-ratio coordinates and not raw concentrations, so unlike traditional correlations that reflect covariance in absolute values, here they describe associations in terms of relative dominance within the composition.

Next, Rondelli and colleagues performed *variance analyses*. Their ANOVA results showed no significant compositional differences, either between the inner and outer areas of the house or among specific activity zones (Rondelli *et al*., 2014: 487). As with correlation analysis, however, applying ANOVA directly to raw compositional data is inappropriate, since the constant-sum constraint unavoidably produces spurious results that obscure true variation. Following the guidelines of Martín-Fernández *et al*. (2015b), group comparisons in a CoDA framework should instead be carried out on log-ratio coordinates applying MANOVA, since the aim is to test differences within the entire compositions rather than in a single log-ratio variable and classical ANOVA can only evaluate univariate contrasts. When MANOVA’s normality and homoscedasticity assumptions are not met, distance-based methods such as perMANOVA on Aitchison distances are recommended (Martín-Fernández *et al*., 2015b: 250). Using the latter, we detected statistically significant differences between the veranda and the interior of the house that were not identified by Rondelli *et al*. (2014). Still, the effect size is modest (ca. 4% of the total variance), indicating that the separation between these contexts is relatively weak in compositional terms.

Finally, regarding *spatial analysis,* Rondelli *et al*. (2014) combined principal component analysis (PCA) with Inverse Distance Weighting (IDW) to map anthropic activity areas derived from element groupings previously proposed in the literature (*e.g*., Ca + Sr for food remains; P + K for burning areas; see Rondelli *et al*. 487–488). PCA scores mapped with IDW treat elemental concentrations as independent variables in the Euclidean space. Because of data compositionality, such procedures are vulnerable to spurious correlations, causing geostatistical interpolation to propagate incoherent cross-structures that violate subcompositional coherence (Pawlowsky-Glahn & Egozcue, 2016; Tolosana-Delgado & Mueller, 2021). Further, relying on previously established combinations of elements while using absolute concentration values also assumes that these can be meaningfully compared across sites, ignoring differences in geological composition and taphonomic history. A CoDA-consistent alternative would be to encode a priori hypothetical groups of elements as log-ratio balances rather than using additive chemical indices. Such a solution would be the ideal end-state of a CoDA-based AAMs framework, but it requires robust knowledge of site-specific background chemistry and well-characterized activity signatures in order to select meaningful log-ratio relationships and avoid circular interpretation of the geochemical signatures. Until such reference models become available, we propose a simpler, data-driven workflow that uses log-ratio coordinates to generate geostatistical elemental maps by applying kriging techniques—in this case, cokriging.

Compared to IDW—which estimates values from distance-weighted neighbours without modelling spatial dependence— cokriging explicitly represents the spatial continuity of each coordinate, taking into account both spatial autocorrelation and elements’ cross-covariances, while also providing uncertainty quantification (see Tolosana-Delgado *et al*., 2019; Tolosana-Delgado and Muller, 2021). To do so, kriging techniques rely on a spatial continuity model that is independent of sample spacing but defined for all lags. In the univariate case, this is a Linear Model of Regionalisation (LMR), which represents variability as a sum of structures acting at distinct spatial scales (Wackernagel, 2003). With multivariate compositional data, we instead fit a Linear Model of Coregionalisation (LMC), which extends the LMR by jointly modelling direct and cross-variograms of the log-ratio coordinates: once fitted, the LMC supports spatial prediction by cokriging, which interpolates multiple variables simultaneously using the spatial dependencies captured by the LMC to assign weights to observations at sampled locations. These weights reflect both the direct spatial continuity of each variable and their cross-correlations and hence are used to provide predictions at unsampled locations (Wackernagel, 2003).

**Fig. 5:**
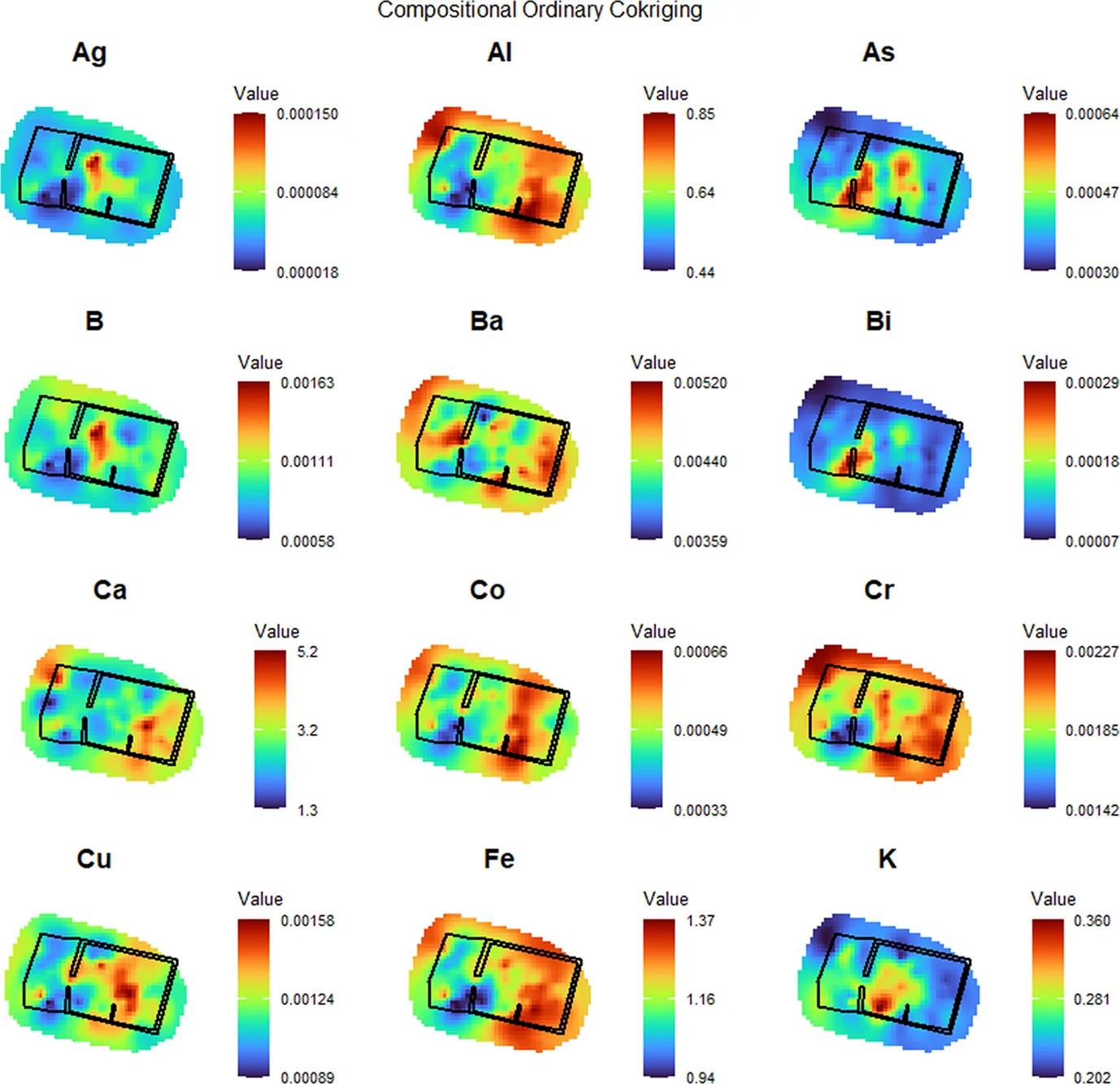

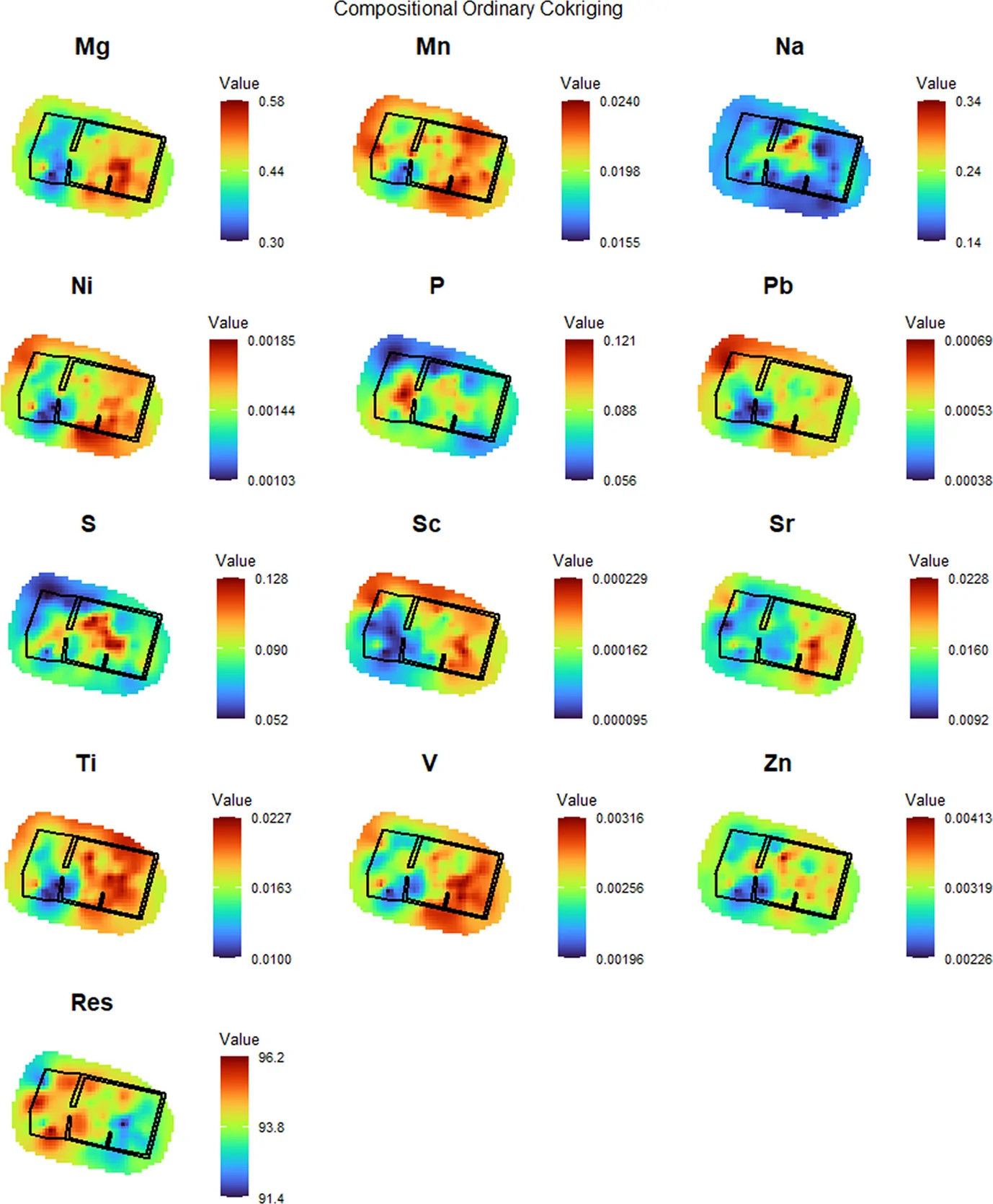
Cokriging elemental maps of *House A* from Jandhala, northern Gujarat. All modelled elements are presented to emphasise the compositional nature of the dataset. Restricting the representation to a subset of elements would reinforce a single-element narrative, obscuring the relational structure between components that underpins the analysis

This alignment between Aitchison geometry (via olr) and geostatistics (via LMC) not only yields compositionally coherent maps together with formal uncertainty, but it also contributes to building the basis for future reference models by enabling log-ratio balances to be compared across sites with greater confidence despite geological differences. Figure 5 shows the results of cokriging modelling applied to the data from Jandhala. Altogether, the cokriging maps of single elements delineate the various activity areas identified ethnographically, including the separation between the veranda and the interior room, but also the different internal areas of the house evidencing the repetitive use of space over a long period of time —*i.e*., the food production and consumption area in the entrance with the adjacent cooking space, as well as the sleeping and storage areas at the back of the house.

**Fig. 6:**
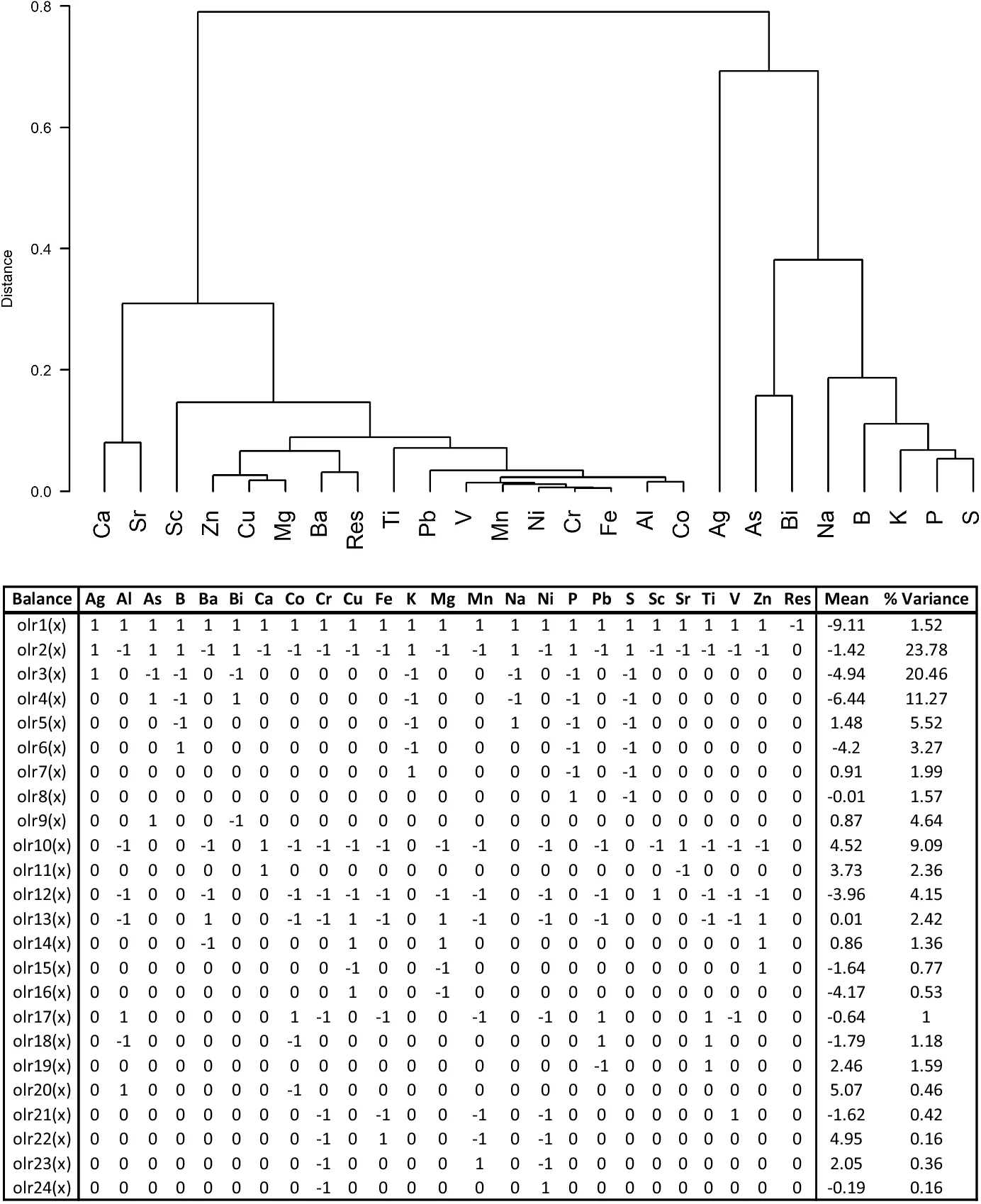
CoDA dendrogram of the Jandhala dataset showing the sequential binary partition used to construct exploratory log-ratio balances

However, the interpretative strength of a compositional approach lies not only in the mapping of individual elements, but also in the analysis of log-ratio relationships between them. Exploratory log-ratio balances were produced using sequential binary partition (SBP) based on dendrogram analysis (Fig. 6, see Egozcue & Pawlowsky-Glahn, 2005; Van Den Boogaart & Tolosana-Delgado, 2013: 90–93 for details; Martínez-Fernandez *et al*., 2018) in order to identify potentially informative contrasts. These log-ratio balances express the contrast between two parts (or groups of parts) of the composition, summarising the dominant geochemical relationships in a single interpretable variable. Because the olr coordinates form an orthogonal basis of the compositional space, each balance is uncorrelated to the others. Consequently, the spatial pattern of any given balance represents that specific contrast orthogonalised with respect to the rest of the composition, ensuring that its variability is not confounded by other elements. This property allows the maps to isolate distinct geochemical processes or activity-related signatures, hence allowing us to identify which log-ratio balances might be relevant to study in each particular case (see Fig. 6).

**Fig. 7:**
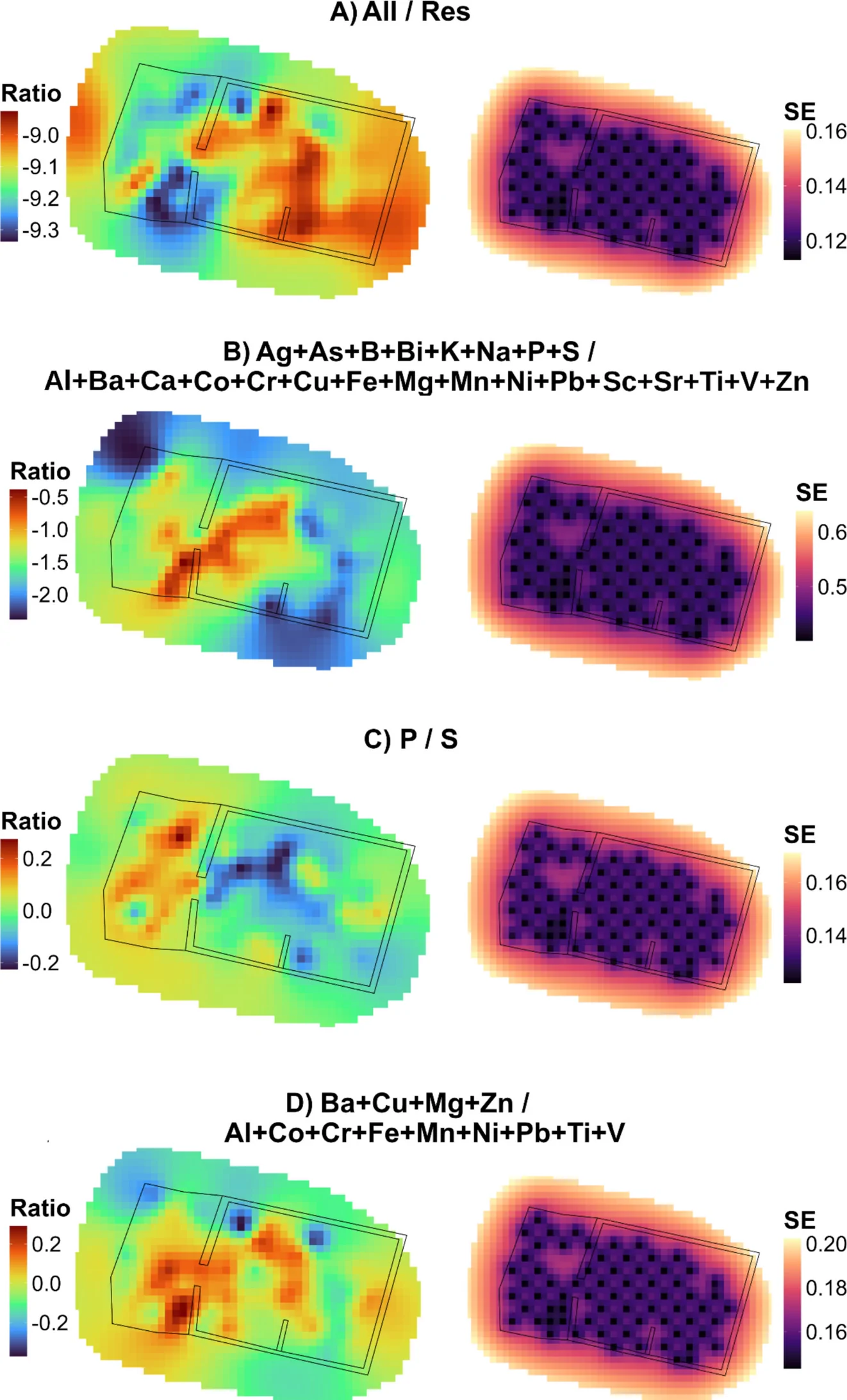
Examples of elemental maps (with uncertainty maps) of useful log-ratio balances from House A (Jandhala, northern Gujarat)

Applied to the Jandhala dataset, these balances isolate distinct spatial contrasts that can be related to specific activity areas and formation processes (see ESM 2, 3, and 4 for detailed results). The following examples illustrate some of the most informative balances identified in this case study. The olr1 balance (Fig. 7a) reflects the relationship between the sum of the identified elemental composition (“All”) and the unmeasured fraction of the dataset (“Res”). It provides a general overview of total measurable elements, highlighting broader spatial differences between activity areas of human frequentation (sleeping and storage areas in addition to food production/consumption) and fireplaces. In this regard, it is possible that the combustion process within fireplaces leads to overall depletion of measurable elements in these areas comparative to all other areas. Olr2 (Figure 7b) contrasts the Ag-As-B-Bi-K-P-Na-S cluster against the rest of the composition, excluding the unmeasured fraction. This balance captures the trade-off between anthropic inputs from organic matter from the food production, cooking and consumption areas and more lithogenic elements of the mineral matrix, which are more prevalent in the sleeping and storage areas in addition to the borders of the veranda. Areas with organic matter inputs can also be observed in the elemental maps of As, Bi, K, P and S (Fig. 5). Similar distribution patterns can be seen between K, Na, P and S (Fig. 5) related to the food production and consumption areas, consistent with previous explanations of the origin of these elements (Misarti *et al*., 2011; Save *et al*., 2020; Wilson *et al*., 2008, 2009). Moreover, lithogenic elements like Al and Fe (Fig. 5) tend to appear depleted in these same areas that seem to be characterized by organic matter inputs. Noted that these elements act as indirect indicators of activity areas when found in depleted values in comparison to other elements like high P. Keeping in mind the compositional nature of multielemental data, this phenomenon is explained by the aforementioned trade-off. The P/S balance (olr8) in Fig. 7c isolates the relative dominance between phosphorus and sulphur. From a geochemical perspective, differences in the preservation and mobility of these elements, linked to organic matter inputs, redox conditions, and/or post-depositional transformation, may contribute to their contrasting spatial distributions: in the present case, the observed pattern likely reflects differential accumulation and transformation of organic residues between interior and exterior spaces (Misarti *et al*., 2011; Oonk *et al*., 2009a; Save *et al*., 2020; Wilson *et al*., 2008, 2009). Finally, olr13, the Ba-Cu-Mg-Zn to Al-Co-Cr-Fe–Mn-Ni-Pb-Ti-V balance (Fig. 7d), can help to differentiate between the fireplace in the veranda and the two internal fireplaces. This distinction aligns with ethnoarchaeological observations by Rondelli *et al*. (2014), who noted that the veranda fireplace was fuelled predominantly with dung, while the internal ones relied mainly on wood. It was also observed that the interior area was regularly cleaned—including the hearths—whereas the exterior was not. Differences between the two types of fireplaces can also be observed in Fig. 7b, as well as in some single element maps in Fig. 5, indicating higher ratios of Bi, K, Na, P, S, and also As in the measurements of the veranda fireplace. The indoor fireplaces, in contrast, show relatively lower concentrations of these same anthropogenic and biogenic elements. These contrasts may reflect differences in fuel type or cleaning practices. From a formation-process perspective, such variability is consistent with known differences in ash composition and elemental retention between dung- and wood-derived fuels, which are influenced by factors such as combustion temperature, mineral content, and post-depositional alteration (*e.g*., Braadbaart *et al*., 2012; Canti, 2003; Lisetskii & Stolba, 2022). However, given the equifinality inherent to these processes, both behavioural and taphonomic explanations remain plausible in this case. Additional research will be needed to better assess the relative contribution of these factors.

Together, these maps are an example of how a CoDA-based geostatistical approach can reveal coherent spatial patterning in anthropogenic sediments without relying on absolute concentrations or pre-defined elemental indices. By expressing geochemical variability as orthonormal log-ratio balances, cokriging provides a compositionally coherent representation of how different geochemical domains interact in space, integrating both elemental and contextual information within a unified statistical framework. Still, the mapped log-ratio balances presented in Fig. 7 were identified through cluster analysis, and hence are specific to the case study. This means that they cannot be directly applied to other sites, as their interpretation depends on local geochemical and taphonomic conditions. Rather, they illustrate how log-ratio balances can be used to generate interpretable signals that integrate chemistry with spatial context. In this sense, the systematic collection and analysis of samples from experimental and ethnoarchaeological contexts is crucial for constructing robust reference models. Similarly, background samples can provide the baseline compositional variability of local sediments (and, when identifiable, of construction materials derived from them) against which anthropogenic enrichments can be measured. By characterizing the general geochemical signal of a site, background and control samples can help disentangle anthropogenic site formation processes from specific activity-related signals. Moreover, they allow log-ratio balances to be contextualized in terms of both local geology and taphonomic history of each site, thereby improving comparability across sites. As more well-documented datasets become available, they will serve as anchors for selecting meaningful log-ratio balances, progressively enabling a more standardized and transferable CoDA-based framework for AAMs analysis.

## Concluding Remarks

Over the past decade, AAMs studies have moved from an exploratory stage into a structured inferential framework, linking biological and geochemical signatures with human behaviour. This has marked an important shift where multivariate and spatial analyses are increasingly favoured over single-element heuristics, demonstrating that AAMs can capture the diversity of uses of space even when material evidence is limited or absent. A key advancement in interpretative terms has been to acknowledge that geochemical datasets are inherently compositional in nature. While ratios and log-ratio expressions are standard practice in geochemistry (see Zuo *et al*., 2021), their application within a formal compositional framework remains limited in AAM research (but see Ginau *et al*., 2020; Biagetti *et al*., 2021; 2026; Danielisová *et al*., 2022; Holmqvist & Ilves, 2022; Janovský *et al*., 2022; Horák *et al*., 2023). When element concentrations are treated as independent variables, analysis risks reproducing statistical artefacts of closure rather than meaningful evidence of human activity. A CoDA-informed framework, by contrast, recognises that these datasets are relative rather than absolute, reframing variation in terms of ratios hence allowing archaeologists to exploit the full potential of data-driven multivariate geochemistry.

Despite the limited number of geoarchaeological studies employing CoDA, the application of this approach is not new to archaeology, especially in the field of archaeometry, where there has been discussion about the implementation of a compositional approach since the 1990s (see Tangri & Wright, 1993; Aitchison *et al*., 2002; Baxter *et al*., 2005; Baxter & Freestone, 2006; Baxter, 2008, 2015; Buxeda i Garrigós, 2008; Martín-Fernández *et al*., 2015a; Wood & Greenacre, 2021). In fact, recent efforts have been made to provide accessible introductions to CoDA for archaeologists (see Martín-Fernández *et al*., 2015a; Greenacre & Wood, 2024). At the same time, some authors have argued against the use of log-ratio transformations in archaeological analysis (Baxter, 2008, 2015; Baxter & Freestone, 2006; Baxter *et al*., 2005), suggesting that regardless of mathematical theory, “[…] *the simpler (if not necessarily ‘theoretically correct’) methods that are prevalent in the literature* […]” are often more effective, as they produce results easier to interpret archaeologically (Baxter, 2015: 111, see also Baxter & Freestone, 2006). While it is true that approaches based on compositional data analysis have limitations in terms of interpretability, this is a result of the mathematical properties of the data and not a flaw of the methodology itself (see Buxeda i Garrigós, 2008). The challenge lies in that archaeological data is often complex, noisy, and incomplete. We argue that the solution should be based on adapting our research questions to align with the nature and structure of data, taking into account not only its potential but also its limitations. In doing so, it is possible to obtain more robust, scientifically sound interpretations that are archaeologically relevant despite the more complex interpretative process. In this sense, mathematical correctness should not be viewed as a barrier, but as a pathway to deeper and more accurate interpretations.

In this paper, we have demonstrated a fully compositionally coherent workflow for AAMs analysis, emphasising two main contributions: first, the systematic use of orthonormal log-ratio balances instead of ad hoc ratio selection; and second, the integration of compositionally coherent geostatistical modelling through cokriging based on LMCs. More broadly, the adoption of a CoDA framework in AAM studies opens a path towards cumulative knowledge-building. Using this approach, spatial models can potentially be compared across contexts, provided that the studies systematically report background samples and model parameters. As noted above, the absence of background and control samples represents the principal limitation of the present study. Without independent characterisation of natural soils and construction materials, it is not possible to establish an absolute baseline against which anthropogenic enrichment or depletion can be assessed. Consequently, while the compositional patterns identified here clearly differentiate activity areas, their interpretation remains relative and does not allow us to unambiguously attribute observed contrasts to specific anthropic inputs as opposed to variability in substrate composition or construction practices. Future applications of this framework should therefore systematically include background samples from local soils, samples of construction materials, and/or control samples from unused or minimally used contexts, depending on the objectives of the analysis. In doing so, results from different projects may be brought into a dialogue, moving AAM research from site-specific pattern recognition towards transferable reference frameworks. This is precisely the direction being pursued within the CAMP project (see Biagetti *et al*. 2026), which will extend the presented approach to new ethnoarchaeological contexts, focusing on how everyday activities perturbate the baseline geochemical composition of human settlements in order to build robust models that can be then compared against archaeological settings. The challenge ahead is therefore not only technical, but conceptual: to rethink what counts as a “marker”, and to accept that AAMs are best understood as spatially structured relational signals rather than fixed elemental thresholds. If multivariate reasoning was the first necessary step, then applying compositional framework is the next one that should shape the path forward.

Finally, it is worth noting that the adoption of CoDA does not in itself resolve the interpretative challenges inherent to archaeological geochemistry. In particular, issues of equifinality and formation processes remain central. The compositional structure identified through CoDA reflects the combined effects of anthropic inputs, depositional conditions, and post-depositional alteration. Processes such as leaching, organic matter decomposition, combustion, and mineral transformation can modify elemental relationships in ways that are not always straightforward to disentangle. While log-ratio approaches provide a statistically coherent representation of these relationships, understanding their archaeological significance still requires explicit engagement with taphonomic and geochemical formation processes. In this sense, CoDA should be understood as a data-driven tool that constrains the analytical space (ensuring that identified patterns are not statistical artefacts) rather than as a substitute for process-based interpretation. Importantly, this does not imply abandoning the theoretically guided approaches that have traditionally structured archaeological research. While selecting variables (and ratios) a priori in relation to specific research questions has proven effective, it also limits the exploration of alternative relationships in increasingly complex, high-dimensional datasets such as multi-elemental geochemical data. In fact, recent work in the philosophy and methodology of science suggests that strongly theory-motivated data collection may bias sampling towards observations that are easier to accommodate within existing frameworks, potentially hindering the development of more accurate or generalisable representations of the system (Dubova *et al*., 2026). In this sense, CoDA not only provides a statistically coherent way of analysing compositional data, but also creates the conditions for more reliable archaeological interpretation. By identifying meaningful relational patterns before assigning them interpretative value, it strengthens existing theoretical frameworks and supports a more rigorous development of AAM research.

## Supplementary Information

Below is the link to the electronic supplementary material.

ESM 1 - Glossary of Compositional Data Analysis Concepts (DOCX 30.9 KB)

ESM 2 - R code of the presented analysis (ZIP 72.1 KB)

ESM 3 - Extended results in HTML (HTML 21.3 MB)

ESM 4 - Extended results in PDF (PDF 22.0 MB)

ESM 5 - Raw data (XLS 50.0 KB)

## Data Availability

The geochemical dataset re-analysed in this study is available as supplementary material to Rondelli *et al*. (2014) at [https://doi.org/10.1016/j.jas.2013.09.008] (https://doi.org/10.1016/j.jas.2013.09.008). The R code employed to perform compositional analysis is available at [https://github.com/project-camp/camp-public] (https:/github.com/project-camp/camp-public).
