# Supplementary material for: Anthropic Activity Markers 2.0: A Shift Towards Compositional Data Analysis
Source: J Archaeol Method Theory. 2026 Jun 10;33(4):64. doi: 10.1007/s10816-026-09799-9 (PMC13249643; doi:10.1007/s10816-026-09799-9)
Supplement: Supplementary file 1 — (DOCX 30.9 KB) [file 10816_2026_9799_MOESM1_ESM.docx]

**Anthropic Activity Markers 2.0: a shift towards compositional data analysis**

Abel Ruiz-Giralt (1, *), Stefano Biagetti (1, 2, 3), Carla Lancelotti (1, 2), Antonios Koutroumpas (1), Keelie S. Rix (1), Jordi Ibañez-Insa (4), Marco Madella (1, 2, 3).

(1) CASEs Research Group, Universitat Pompeu Fabra. C. Ramon Trias Fargas 25-27, 08005, Barcelona, Spain

(2) ICREA, Passeig Lluís Companys 23, Barcelona, Spain

(3) School of Geography, Archaeology and Environmental Studies (GAES), University of the Witwatersrand, 1 Jan Smuts Avenue, Braamfontein 2000, Johannesburg, South Africa

(4) Geosciences Barcelona (GEO3BCN-CSIC), Lluís Solé i Sabarís s/n, 08028, Barcelona, Spain

(*) Corresponding author: [abel.ruiz@upf.edu](mailto:abel.ruiz@upf.edu)

**Supplementary Materials 1 (SM1): Glossary of Compositional Data Analysis Concepts**

This supplementary section provides a concise glossary of the main concepts, definitions, and mathematical terms used throughout the paper Anthropic Activity Markers 2.0: A shift towards compositional data analysis. Its purpose is to facilitate the interpretation of the methodological framework and ensure conceptual clarity for readers less familiar with the principles of Compositional Data Analysis (CoDA).

**The simplex geometry:** A composition is a vector of positive components, 𝑥=[𝑥1​,𝑥2​,...,𝑥𝐷​], where every component is called *part* of the composition. In the simplex space, $S^{D},$ the sum of the parts of a composition is a constant *k,* where:

$$\sum_{i=1}^{D} x_{i}= k$$

The constant k is typically set to 1 for proportions or 100 for percentages. This constant-sum property, called *closure* or *normalization* (Aitchison, 1986; Greenacre & Wood, 2024), creates the fundamental interdependence within the parts of the composition: because the total is fixed, an increase of one part necessarily results in a proportional decrease of one or more parts of the composition. Consequently, the absolute values of the parts can be misleading; only the relative information—the relationships between the parts—is relevant for analysis. This interrelationship trait implies that the sample space $S^{D},$ of all D-part compositions is effectively a D-1-dimensional space, rather than an Euclidean D-dimensional one, since once the D-1 parts are defined, the final part is automatically determined due to its closure property, resulting in D-1 degrees of freedom. Bearing this, a three-part composition (𝐷=3) which has only 2 degrees of freedom (𝐷-1=2) can be fully represented on a two-dimensional ternary diagram, where the sum of perpendicular distances from any point to the triangle's sides remains constant, mirroring the compositional constraint itself.

## **Equivalence classes of compositions:** As was described above, the core principle of compositional analysis is the analysis of the relative information within a composition, not the absolute values of parts. Therefore, compositions that share the same internal relative information are considered equivalent, regardless of their total magnitudes. This concept groups such compositions into what is known as an *equivalence class*. Mathematically, two compositions **x** and **y** are compositionally equivalent if one is a scalar multiple of the other:

𝑦 = a𝑥 with a > 0 positive constant

## For example, the compositions [30, 20, 50] and [3, 2, 5] represent identical relative proportions (30%, 20%, 50%) despite having different absolute magnitudes. This property leads to a critical requirement in compositional analysis: *scale invariance,* meaning that it must produce identical results for all members of an equivalence class, ensuring that the analysis is based on the ratios between parts (Barceló‐Vidal, 2016).

## **Aitchison geometry operations:** The Aitchison geometry defines a rich algebra on the simplex space $S^{D}$ through two fundamental operations that respect the compositional nature of the data, enabling formal statistical operations.

1. **Perturbation** (⊕) is the compositional equivalent of vector addition in Euclidean space. Given two compositions 𝑥 = [𝑥₁, ..., 𝑥𝐷] and 𝑦 = [𝑦₁, ..., 𝑦𝐷] in $S^{D}$, their perturbation is defined as:

$$x \oplus y = C[x_{1}y_{1},x_{2}y_{2}, ..., x_{D}y_{D}]$$

where C denotes the closure operation. It combines two compositions by multiplying their corresponding parts and then re-closing (normalizing) the result to ensure it sums to the required constant. Geometrically, perturbing a composition by **y** is like "translating" or shifting the composition x within the simplex according to the proportions in y. This operation is commutative (**x** ⊕ **y** = **y** ⊕ **x**) and associative, and contains a neutral element, 𝑛 = [1/𝐷, ..., 1/𝐷], which acts like zero in standard addition: **x** ⊕ **n** = **x**. This **neutral element**, often called the center of the simplex, is a **fixed theoretical point**, representing a composition of perfect uniformity where all parts are equal (Aitchison & Ng, 2005).

1. **Powering** (⊙) is the compositional analogue of scalar multiplication. It scales a composition x by a real number α by raising each part to the power of α and then applying closure.

$$\alpha\odot x=C[x_{1}^{a}, x_{2}^{a}, ..., x_{D}^{a}]$$

Powering scales a composition by raising each component to the power α and then applying closure, effectively stretching or shrinking the composition relative to the center of the simplex. When α > 1, powering amplifies the relative differences between parts; when 0 < α < 1, it mutes the differences, pulling the composition closer to the center; and when α = 0, it transforms the composition into the neutral element **n**. This operation satisfies properties analogous to scalar multiplication: (α + β) ⊙ **x** = (α ⊙ **x**) ⊕ (β ⊙ **x**) and α ⊙ (**x** ⊕ **y**) = (α ⊙ **x**) ⊕ (α ⊙ **y**).

Together, perturbation and powering provide the simplex with a vector space structure, making it possible to perform operations parallel to standard linear algebra while respecting the constraints of compositional data (Aitchison, 1986).

## **Principles of compositional data analysis:** Taking into account that compositions show relative proportions of parts within a whole, not absolute quantities, the analysis of compositional data is based on several essential principles. The first one, called the principle of **scale invariance**, highlights that the absolute size of a composition is irrelevant. Basically, what is taken into account are the relative amounts, not the absolute quantities; thus, multiplying all parts by the same positive constant doesn't affect the compositional information, formally expressed as f(k**w**) = f(**w**) for k > 0, where f represents any valid compositional analysis function (Aitchison, 1986; Barceló‐Vidal and Fernández, 2016).

Within the same context, compositional data follow the **permutation invariance** principle, which states that rearranging the parts of a composition doesn't change the statistical results. For example, the distance between two soil compositions should be identical whether analyzing [sand, clay, silt] or [clay, sand, silt], and removing different components to avoid mathematical singularity should yield equivalent outcomes—though classical methods that simply delete one component violate this principle since results depend heavily on which component is removed (Aitchison, 1986).

Finally, **subcompositional coherence** states that the analysis of a part of a composition, called a subcomposition, should yield the same conclusions as analyzing the same components within the complete composition. This consistency is maintained because the log-ratios between components—the fundamental information in compositional data—remain unchanged whether analyzing a subcomposition or the full composition (Aitchison, 1986).

## **Log-ratio transformations:** Considering the unique geometry of the simplex and the necessity to respect the principles mentioned above, logarithmic transformations are introduced within compositional data analysis to move the data from the simplex to Euclidean space, converting ratios into logarithms and removing the closure effect (Aitchison, 1986; Egozcue and Pawlowsky-Glahn, 2011). Fundamentally, these transformations map the relationships between components from the constrained simplex space into unconstrained real space, where standard multivariate statistical methods can be applied.

### **Additive log-ratio (alr) transformation:** The **additive log-ratio (alr) transformation** provides a straightforward approach to mapping compositional data from the simplex to real space. Given a D-part composition **x** = [x₁, ..., $x_{D}$] ∈ $S^{D}$, the alr transformation maps it to a (D-1)-dimensional real vector by taking logarithms of ratios between components. Specifically, each of the first D-1 components is divided, usually, by the final component $x_{D}$, which serves as a common reference:

𝑎𝑙𝑟(𝑥) = [𝑙𝑛($x_{1}$/$x_{D}$), 𝑙𝑛($x_{2}$/$x_{D}$), ..., 𝑙𝑛($x_{D-1}$/$x_{D}$)]

This transformation is one-to-one, allowing for back-transformation through:

𝑎𝑙𝑟⁻¹(𝑦) = 𝐶[𝑒𝑥𝑝($y_{1}$), 𝑒𝑥𝑝($y_{2}$), ..., 𝑒𝑥𝑝($y_{D-1}$), 1]

where C denotes the closure operation that ensures the result sums to the constant κ.

While the alr transformation successfully maps compositions to unconstrained ℝ^D-1^ space, its asymmetric treatment of parts and, more critically, its failure to preserve distances—meaning the Aitchison distance between compositions does not equal the Euclidean distance between their alr-transformed counterparts—can distort geometric relationships when applying standard statistical methods.

### **Centered log-ratio (clr) transformation:** The **centered log-ratio (clr) transformation** treats all parts symmetrically by dividing each component by the geometric mean of all components before taking logarithms. For a D-part composition, the transformation produces a D-dimensional vector where each element is the natural logarithm of the ratio between that component and the geometric mean:

𝑐𝑙𝑟(𝑥) = [𝑙𝑛($x_{1}$/𝑔(𝑥)), 𝑙𝑛($x_{2}$/𝑔(𝑥)), ..., 𝑙𝑛($x_{D}$/𝑔(𝑥))]

where 𝑔(𝑥) = (𝑥₁ · 𝑥₂ · ... · $x_{D}$)^(1/𝐷)^ is the geometric mean of the components.

The clr transformation maps the simplex to a (D-1)-dimensional subspace of real D-dimensional space. The transformation is one-to-one, with the inverse achieved through exponentiation followed by closure:

𝑐𝑙𝑟⁻¹(𝑧) = 𝐶[𝑒𝑥𝑝($z_{1}$), 𝑒𝑥𝑝($z_{2}$), ..., 𝑒𝑥𝑝($z_{D}$)]

Subsequently, the clr transformation is an isometry, preserving Aitchison distances between compositions—a property essential for metric-dependent analyses. However, clr-transformed vectors are constrained to sum to zero, resulting in a singular covariance matrix, making correlation coefficients uninformative and certain multivariate methods inapplicable (Filzmoser et al., 2009; Filzmoser et al., 2010).

### **Orthonormal log-ratio (olr/ilr) transformation:** To overcome these limitations, the **olr transformation**, also known as the **isometric log-ratio (ilr) transformation**, was developed, providing orthonormal coordinates in (D-1)-dimensional real space. For a composition in the simplex and an orthonormal basis consisting of D-1 compositions, the olr transformation produces coordinates by computing the Aitchison inner product between the composition and each basis element:

𝑜𝑙𝑟(𝑥) = [⟨𝑥, 𝑒₁⟩_A_ ..., ⟨𝑥, 𝑒_𝐷-1_⟩_𝐴_]

where ⟨·, ·⟩𝐴 denotes the Aitchison inner product.

The basis compositions must satisfy orthonormality conditions (unit Aitchison norm and mutual orthogonality) to preserve geometric properties, with each resulting coordinate representing an orthogonal projection interpretable as log-contrasts or balances between component groups. To elaborate on this, a log-contrast is a logarithm of a ratio of geometric means of groups of components, or equivalently, a linear combination of log-transformed components with coefficients that sum to zero. This transformation achieves both isometry and isomorphism, preserving distances while providing a one-to-one mapping between the simplex and ℝ^D-1^ (Egozcue et al., 2003). The transformation is fully reversible, allowing movement between the simplex and coordinate space in both directions. Different orthonormal bases can be constructed, such as through sequential binary partitions, providing flexibility in interpretation while maintaining all mathematical properties. Critically, all compositional operations (perturbation and powering) and metric relationships in the simplex translate directly to standard vector operations (addition and scalar multiplication) and Euclidean distances in the coordinate space, enabling the unrestricted application of conventional statistical methods without geometric distortion.

**Symmetric balances:** While the olr/ilr transformation provides orthonormal coordinates suitable for standard statistical analysis, correlation analysis between specific compositional parts requires careful consideration. Traditional approaches of simply correlating individual parts or even clr coefficients can produce misleading results due to the negative bias inherent in the constrained covariance structure. Symmetric balances offer a geometrically sound solution for measuring associations between two compositional parts of interest (Kynčlová et al., 2017). For parts xi and xj, symmetric balances are constructed by creating coordinates that express each part's dominance relative to a weighted geometric mean of all other parts, treating both parts symmetrically within the coordinate system.

Symmetric balance for part i (relative to part j):

$$z_{i}^{s}=\sqrt{\frac{D-1+\sqrt{D(D-2)}}{2D}}ln\frac{x_{i}}{x_{j}^{a}{{(\prod_{k\neq i,j}^{D} x_{k})}^{\beta}}}$$

$z_{j}^{s}=\sqrt{\frac{D-1+\sqrt{D(D-2)}}{2D}}ln\frac{x_{j}}{x_{i}^{a}{(\prod_{k\neq i,j}^{D} x_{k})}^{\beta}}$,

where the exponents α and β are determined to ensure orthonormality while maintaining symmetry between the two parts of interest. These coordinates capture how each part dominates relative to the "average representative" of the remaining parts and are defined by the following

$a=\frac{1}{D-1+\sqrt{D(D-2)}}$, $\beta=\frac{\sqrt{D-2}+\sqrt{D}}{\sqrt{D-2}(D-1+\sqrt{D(D-2)})}$

The Pearson correlation coefficient between symmetric balances provides an interpretable measure where positive correlation indicates that both parts increase their relative dominance simultaneously, negative correlation suggests opposing dominance patterns, and near-zero correlation implies independent processes controlling each part's behavior. Importantly, this approach respects subcompositional coherence: the correlation reflects the relationship within the specific compositional context being analyzed, appropriately accounting for all parts present. This makes symmetric balances particularly valuable for identifying underlying processes in geochemical, ecological, and other compositional datasets where understanding pairwise associations is essential for scientific interpretation.

## **The centre of a composition** is the analogue of the "average composition" in Euclidean space. Calculating a simple average, the arithmetic mean, for compositional data is misleading because it ignores the relative nature of the data and the geometry of the simplex. The use of the geometric mean ensures consistency with the relative (ratio-based) nature of the data, as opposed to the arithmetic mean, which often behaves like an outlier in the simplex. The appropriate measure of central tendency is the **closed geometric mean**. For a dataset X of n observations and D parts, the centre (g) is defined as the closure of the vector of geometric means of the components:

$$\boldsymbol{g}_{\boldsymbol{i}}\boldsymbol{=}\left( \prod_{\boldsymbol{i}\boldsymbol{=1}}^{\boldsymbol{n}} \boldsymbol{x}_{\boldsymbol{ij}} \right)^{\frac{\boldsymbol{1}}{\boldsymbol{n}}}$$

Geometrically, the center is the point that minimizes the total **Aitchison distance** to all other compositions in the dataset, making it the true *middle* of the data cloud. It acts as the natural estimator for the average composition and, unlike the arithmetic mean, fully respects the ratio-based structure of the compositional data (Aitchison, 1986).

### Likewise, the special treatment of “average” (center) in compositional data, the concept of spread or variability of the values, also requires a different approach. The mathematically traditional standard variance is unsuitable, since it ignores the relative nature of the data. Instead, dispersion is captured by recording the variance of all pairwise log-ratios between parts.

**Variation matrix** (**T** = [τᵢⱼ]) is a comprehensive map of the dataset’s internal variability. Each element of this matrix, $t_{ij}$, captures the variance of the log-ratio between part i and part j.

$$t_{ij}=var\left( ln\left( \frac{x_{i}}{x_{j}} \right) \right)$$

A large value for $t_{ij}$ indicates that the relationship between parts i and j is highly variable across the samples, while a small value indicates a more stable, proportional relationship. The matrix is symmetric ($t_{ij}$​=$t_{ji}$​), has zeros on its diagonal ($t_{ii}$=0), and is equivalent to the centered log-ratio (clr) covariance matrix (**Γ**); either can be derived from the other through simple matrix operations (Aitchison, 1986).

**Total variance** summarizes the overall relative variability of the entire compositional dataset into a single number. It is calculated from the entries of the variation matrix.

$$totVar[X] = \frac{1}{2D}\sum_{i=1}^{D} \sum_{j=1}^{D} t_{ij}$$

Together, the **center**, **variation matrix**, and **total variance** form the foundation of descriptive statistics for compositional data. They provide measures of central tendency and dispersion that are mathematically consistent with the geometry of the simplex and the core principles of scale and perturbation invariance(Aitchison, 1986).

Utilization of Aitchison geometry allows the performance of rigorous statistical analysis of clustering or hypothesis testing, allowing the measurement of the “distance” between compositions, analogous to how the Euclidean distances and angles are calculated in $R^{D}$(Aitchison, 1992).

**Aitchison distance** $d_{a}(x,y)$ is the distance metric for measuring the difference between two compositions, x and y, equivalent to the standard Euclidean distance between clr-transformed compositions, effectively unwrapping the “simplex” into the “Euclidean” space (Aitchison, 1992).

$$d_{a}(x,y) = EuclideanDistance(clr(x),clr(y))$$

The properties of this metric align with the principles of compositional data, including:

- **Scale invariance**: The distance between [10,20,70] and [20,30,50] is identical to the distance between [1,2,7] and [2,3,5], ensuring results are independent of the constant sum constraint.
- **Perturbation Invariance:** Two compositions can be “shifted” by perturbing (⊕) them with a third composition, preserving the distances under translation.
- **Subcompositional Coherence:** The distance calculated using only a subset of parts is always less than or equal to the distance calculated using the full composition, ensuring consistent results whether the whole composition or a subpart of it is analysed.

The **Aitchison norm** ($\left| \left| \cdot\right| \right|_{a}$) measures the "length" of a single composition x. This length is defined as its Aitchison distance from the **center** of the simplex (the neutral element, n). A composition with a large norm is far from the center, indicating strong relative differences between its parts, while a composition with a small norm is close to the uniform center (Pawlowsky-Glahn & Egozcue, 2001).

$$\left| \left| x \right| \right|_{a}=\left| \left| clr(x) \right| \right|_{E}$$

The **Aitchison inner product** (${\langle\cdot,\cdot\rangle}_{a}$) defines the geometric relationship between two compositions **x** and **y**, analogous to how the dot product relates to the angle between vectors in standard space. This leads to the definition of compositional orthogonality, when two compositions are perpendicular, having their inner product zero. The definition of this inner product is crucial since it’s equivalent to the standard inner product of the clr-transformed compositions (Pawlowsky-Glahn & Egozcue, 2001).

Along with the operations of perturbation and powering, the establishment of the $S^{D}$ simplex as a Euclidean space of D-1 dimension is allowed, enabling the application of standard multivariate statistical techniques on the log-ratio-transformed data, respecting the underlying constraints of the original compositional data.

**Funding**

This research is supported by the project CAMP funded by the European Union (ERC CoG 2022, CAMP-101088842). The original data was collected as part of the NoGAP project funded by the Spanish Ministry of Science and Innovation (HAR2010-16052) and by the Spanish Ministry of Education, Culture and Sport (Proyectos Arqueológicos en el Exterior). Views and opinions expressed are however those of the author(s) only and do not necessarily reflect those of the European Union or the European Research Council Executive Agency nor any other agency. Neither the European Union nor the granting authority can be held responsible for them.

**Competing Interests**

The authors have no competing interests to declare that are relevant to the content of this article.

**Reference list**

Aitchison, J. (1986). *The Statistical Analysis of Compositional Data*. Dordrecht: Springer Netherlands.<https://doi.org/10.1007/978-94-009-4109-0>

Aitchison, J, & Ng, K. W. (2005). The role of perturbation in compositional data analysis. *Statistical Modelling*, *5*(2), 173–185.<https://doi.org/10.1191/1471082X05st091oa>

Aitchison, John. (1992). On criteria for measures of compositional difference. *Mathematical Geology*, *24*(4), 365–379.<https://doi.org/10.1007/BF00891269>

Barcelo-Vidal, C., & Martín-Fernández, J.-A. (2016). The Mathematics of Compositional Analysis. *Austrian Journal of Statistics*, *45*(4), 57–71.<https://doi.org/10.17713/ajs.v45i4.142>

Egozcue, J. J., & Pawlowsky-Glahn, V. (2011). Basic concepts and procedures. In V. Pawlowsky-Glahn & A. Buccianti (Eds.), *Compositional Data Analysis: Theory and Applications* (pp. 74–91). Hoboken, N.J: Wiley.

Egozcue, J. J., & Pawlowsky-Glahn, V. (2018). Modelling Compositional Data. The Sample Space Approach. In B. S. Daya Sagar, Q. Cheng, & F. Agterberg (Eds.), *Handbook of Mathematical Geosciences: Fifty Years of IAMG* (pp. 81–105). Cham: Springer International Publishing.<https://doi.org/10.1007/978-3-319-78999-6>

Egozcue, J. J., Pawlowsky-Glahn, V., Mateu-Figueras, G., & Barceló-Vidal, C. (2003). Isometric Logratio Transformations for Compositional Data Analysis. *Mathematical Geology*, *35*(3), 279–300.<https://doi.org/10.1023/A:1023818214614>

Filzmoser, P., Hron, K., & Reimann, C. (2009). Principal component analysis for compositional data with outliers. *Environmetrics*, *20*(6), 621–632.<https://doi.org/10.1002/env.966>

Filzmoser, P., Hron, K., & Reimann, C. (2010). The bivariate statistical analysis of environmental (compositional) data. *Science of The Total Environment*, *408*(19), 4230–4238.<https://doi.org/10.1016/j.scitotenv.2010.05.011>

Greenacre, M., & Wood, J. R. (2024). A comprehensive workflow for compositional data analysis in archaeometry, with code in R. *Archaeological and Anthropological Sciences*, *16*(10), 171.<https://doi.org/10.1007/s12520-024-02070-w>

Kynčlová, P., Hron, K., & Filzmoser, P. (2017). Correlation Between Compositional Parts Based on Symmetric Balances. *Mathematical Geosciences*, *49*(6), 777–796.<https://doi.org/10.1007/s11004-016-9669-3>

Pawlowsky-Glahn, V., & Egozcue, J. J. (2001). Geometric approach to statistical analysis on the simplex. *Stochastic Environmental Research and Risk Assessment*, *15*(5), 384–398.<https://doi.org/10.1007/s004770100077>
