# Supplementary material for: Anthropic Activity Markers 2.0: A Shift Towards Compositional Data Analysis
Source: J Archaeol Method Theory. 2026 Jun 10;33(4):64. doi: 10.1007/s10816-026-09799-9 (PMC13249643; doi:10.1007/s10816-026-09799-9)
Supplement: Supplementary file 3 — (HTML 21.3 MB) [file 10816_2026_9799_MOESM3_ESM.html]

 

 

 

 
 
 


 workflow.knit 

 
 
 
 
 
 
 
 
 
 
 
 
 

 

 
 


 


 

 

 


 

 


 


 


 


 
  Anthropic Activity Markers 2.0: a shift towards
compositional data analysis  
 Abel Ruiz-Giralt (1, *), Stefano Biagetti (1, 2, 3), Carla Lancelotti
(1, 2), Óscar Parque (1), Antonios Koutroumpas (1), Keelie S. Rix (1),
Jordi Ibañez-Insa (4), Marco Madella (1, 2, 3) 
 
 CASEs Research Group, Universitat Pompeu Fabra. C. Ramon Trias
Fargas 25–27, 08005, Barcelona, Spain 
 
 ICREA, Passeig Lluís Companys 23, Barcelona, Spain 
 
 School of Geography, Archaeology and Environmental Studies (GAES),
University of the Witwatersrand, 1 Jan Smuts Avenue, Braamfontein 2000,
Johannesburg, South Africa 
 
 Geosciences Barcelona (GEO3BCN-CSIC), Lluís Solé i Sabarís s/n,
08028, Barcelona, Spain 
 
 (*) Corresponding author:  abel.ruiz@upf.edu  
 
  Supplementary Materials 2 (SM2): Results of Compositional
Data Analysis from House A in Jandhala, northern Gujarat  
 This notebook implements the compositional data analysis to the
geochemical dataset by Rondelli et al. (2014) available at  http://dx.doi.org/10.1016/j.jas.2013.09.008 , based on
the workflow developed within the CAMP project (see  https://github.com/project-camp/camp-public ). 
 The packages used include: “stats”, “utils”, “dplyr”, “tidyr”,
“tidyverse”, “magrittr”, “stringr”, “sf”, “sp”, “raster”, “rasterVis”,
“stars”, “gstat”, “spdep”, “automap”,“dbscan”, “grid”, “gridExtra”,
“lattice”, “latticeExtra”, “ggplot2”, “ggdendro”, “ggrepel”,
“ggcorrplot”, “ggpubr”, “colorspace”, “viridis”, “patchwork”, “leaflet”,
“classInt”, “knitr”, “kableExtra”, “readxl”, “jsonlite”, “htmlwidgets”,
“IRdisplay”, “car”, “dendextend”, “MASS”, “MVN”, “RVAideMemoire”,
“vegan”, “NbClust”, “biotools”, “rrcov”, “DescTools”, “compositions”,
“zCompositions”, “robCompositions”, “gmGeostats”. References at listed
at the end of this document 
 
 
 Initial setup and imports 
  # Show code in the rendered document (use echo = FALSE to hide code)
knitr::opts_chunk$set(echo = TRUE)

# Set the default figure size and dpi
knitr::opts_chunk$set(fig.height = 12, fig.width = 18, fig.dpi = 300)

# Hide warnings (to keep the notebook clean)
options(warn = -1) # Set to 0 to show warnings again
knitr::opts_chunk$set(warning = FALSE)

# Set the option to avoid scientific notation
options(scipen = 9)

# Set seed for reproducibility
set.seed(999)

# Import libraries 
required_packages <- c(
  # Base packages
  "stats", "utils",
  # Data manipulation core
  "dplyr", "tidyr", "tidyverse", "magrittr", "stringr", "scales",
  # Spatial packages
  "sf", "sp", "raster", "rasterVis", "stars",  "gstat", "spdep", "automap", 
  "dbscan", "grid", "gridExtra", "lattice", "latticeExtra",
  # Visualization
  "ggplot2", "ggdendro", "ggrepel", "ggcorrplot", "ggpubr", "colorspace", 
  "viridis", "patchwork", "leaflet", "classInt", "knitr", "kableExtra",
  # File I/O
  "readxl", "jsonlite", "htmlwidgets", "IRdisplay",
  # Statistics
  "car", "dendextend", "MASS", "MVN", "RVAideMemoire", "vegan", "NbClust", 
  "biotools", "rrcov", "DescTools",
  # Compositional data
  "compositions", "zCompositions", "robCompositions", "gmGeostats"
)

# Function to check for missing packages and install them automatically
check_missing_packages <- function(packages) {
  missing_packages <- packages[!sapply(packages, requireNamespace, quietly = TRUE)]
  
  if(length(missing_packages) > 0) {
    message("Missing packages that need to be installed: ", paste(missing_packages, collapse = ", "))
    
    # Install the missing packages
    install.packages(missing_packages)
    message("Packages installed successfully.")
  }
  
  # Load all packages silently
  suppressPackageStartupMessages({
    invisible(lapply(packages, library, character.only = TRUE))
  })
  return(TRUE)
}

# Load all packages (and install missing ones automatically)
check_missing_packages(required_packages)  
  ## Registered S3 method overwritten by 'vegan':
##   method     from      
##   rev.hclust dendextend  
  ## Registered S3 method overwritten by 'GGally':
##   method from   
##   +.gg   ggplot2  
  ## Registered S3 method overwritten by 'perry':
##   method        from   
##   print.cvFolds cvTools  
  ## [1] TRUE  
 
 
 Data loading and pre-processing 
  # Load spatial data (house locations) from a GeoJSON file
shp <- st_read("./data/jand_house.geojson")  
  ## Reading layer `jand_house' from data source 
##   `G:\Mi unidad\Archaeology\papers\Anthropic markers 2.0\resubmission\data\jand_house.geojson' 
##   using driver `GeoJSON'
## Simple feature collection with 1 feature and 1 field
## Geometry type: MULTILINESTRING
## Dimension:     XY
## Bounding box:  xmin: 6.9339 ymin: -14.18984 xmax: 17.2185 ymax: -7.526581
## Geodetic CRS:  WGS 84  
  # Load chemical data from an Excel file and show data structure:
data <- read_excel("./data/JandIns_processed.xlsx")
data <- column_to_rownames(data, var = "SAMPLE")
str(data)  
  ## 'data.frame':    70 obs. of  28 variables:
##  $ X       : num  13 12.5 13.5 13.5 13.5 14 13.5 14 14 14 ...
##  $ Y       : num  -11.5 -12 -12 -13 -11 -11.5 -10 -10.5 -13.5 -12.5 ...
##  $ Area    : chr  "Inside" "Inside" "Inside" "Inside" ...
##  $ Activity: chr  "Storage" "Storage" "Storage" "Storage" ...
##  $ Ag      : chr  "0.6" "0.7" "1" "0.4" ...
##  $ Al      : num  7300 6600 7400 8500 5900 7500 7400 9300 8400 8000 ...
##  $ As      : num  5 6 5 6 4 5 7 5 4 5 ...
##  $ B       : chr  "10" "10" "10" "10" ...
##  $ Ba      : num  40 40 50 40 40 40 40 50 40 50 ...
##  $ Bi      : chr  "<2" "<2" "<2" "<2" ...
##  $ Ca      : num  29500 34000 43000 57000 39700 45000 33000 32000 28100 50700 ...
##  $ Co      : num  6 5 6 6 6 6 6 7 9 6 ...
##  $ Cr      : num  20 18 21 21 18 18 19 22 21 19 ...
##  $ Cu      : num  15 13 15 13 14 17 20 18 14 14 ...
##  $ Fe      : num  12100 11800 13000 13600 10400 12700 12300 14300 14100 12700 ...
##  $ K       : num  2800 3200 3100 2100 2700 2700 1800 3300 2100 2700 ...
##  $ Mg      : num  5400 5100 5700 5500 5000 5300 4900 5600 4500 6000 ...
##  $ Mn      : num  217 203 235 223 184 212 224 262 235 227 ...
##  $ Na      : num  2100 1700 2100 1600 2600 2400 500 900 900 2700 ...
##  $ Ni      : num  17 14 16 17 13 17 16 19 20 16 ...
##  $ P       : num  970 960 1090 660 970 990 750 890 450 1160 ...
##  $ Pb      : num  5 5 6 5 4 6 6 6 6 5 ...
##  $ S       : num  1300 900 1200 700 1300 1300 500 700 500 1300 ...
##  $ Sc      : chr  "2" "2" "2" "2" ...
##  $ Sr      : num  175 180 201 194 192 220 211 212 136 245 ...
##  $ Ti      : num  200 200 200 200 200 200 200 200 200 200 ...
##  $ V       : num  27 27 30 34 26 29 28 30 30 31 ...
##  $ Zn      : num  46 31 36 28 32 33 36 41 36 35 ...  
  # Separate group and spatial data
coords <- data[,1:2]
Area <- data[,3]
Activity <- data[,4]

# Convert all columns from the 5th onward to numeric (in case some are stored as text)
data[5:ncol(data)] <- lapply(data[5:ncol(data)], as.numeric)
# Replace missing values (NA) in numeric columns with half the minimum detected value, following Rondelli et al. (2014)
# This is a common approach in geochemistry to handle censored data (below detection limit)
comp <- data[,5:ncol(data)]
comp <- as.data.frame(lapply(comp, function(x) { 
  if(is.numeric(x)) { x[is.na(x)] <- (min(x, na.rm = TRUE)) / 2
  } 
  return(x) 
}))

# Reorder and close compositional object
comp <- comp[, sort(colnames(comp))]
comp$Res <- 1000000 - rowSums(comp, na.rm = TRUE)
str(comp)  
  ## 'data.frame':    70 obs. of  25 variables:
##  $ Ag : num  0.6 0.7 1 0.4 1 1 0.4 0.4 0.2 0.7 ...
##  $ Al : num  7300 6600 7400 8500 5900 7500 7400 9300 8400 8000 ...
##  $ As : num  5 6 5 6 4 5 7 5 4 5 ...
##  $ B  : num  10 10 10 10 10 10 5 10 5 10 ...
##  $ Ba : num  40 40 50 40 40 40 40 50 40 50 ...
##  $ Bi : num  1 1 1 1 1 2 1 1 1 1 ...
##  $ Ca : num  29500 34000 43000 57000 39700 45000 33000 32000 28100 50700 ...
##  $ Co : num  6 5 6 6 6 6 6 7 9 6 ...
##  $ Cr : num  20 18 21 21 18 18 19 22 21 19 ...
##  $ Cu : num  15 13 15 13 14 17 20 18 14 14 ...
##  $ Fe : num  12100 11800 13000 13600 10400 12700 12300 14300 14100 12700 ...
##  $ K  : num  2800 3200 3100 2100 2700 2700 1800 3300 2100 2700 ...
##  $ Mg : num  5400 5100 5700 5500 5000 5300 4900 5600 4500 6000 ...
##  $ Mn : num  217 203 235 223 184 212 224 262 235 227 ...
##  $ Na : num  2100 1700 2100 1600 2600 2400 500 900 900 2700 ...
##  $ Ni : num  17 14 16 17 13 17 16 19 20 16 ...
##  $ P  : num  970 960 1090 660 970 990 750 890 450 1160 ...
##  $ Pb : num  5 5 6 5 4 6 6 6 6 5 ...
##  $ S  : num  1300 900 1200 700 1300 1300 500 700 500 1300 ...
##  $ Sc : num  2 2 2 2 1 2 2 2 2 2 ...
##  $ Sr : num  175 180 201 194 192 220 211 212 136 245 ...
##  $ Ti : num  200 200 200 200 200 200 200 200 200 200 ...
##  $ V  : num  27 27 30 34 26 29 28 30 30 31 ...
##  $ Zn : num  46 31 36 28 32 33 36 41 36 35 ...
##  $ Res: num  937743 934984 922575 909540 930684 ...  
 
 
 Descriptive statistics 
  # Calculate descriptive statistics
descstats <- compositions::summary.acomp(comp)
descstats$totvar <- sum(descstats$variation) / (2 * ncol(comp))

# Print center
cat("Compositional centre (closed to 1)")  
  ## Compositional centre (closed to 1)  
  descstats$mean  
  ##                Ag                Al                As                 B 
## "0.0000005636011" "0.0063981774936" "0.0000043442831" "0.0000097326400" 
##                Ba                Bi                Ca                Co 
## "0.0000442190793" "0.0000012727692" "0.0293684872084" "0.0000049302816" 
##                Cr                Cu                Fe                 K 
## "0.0000190071593" "0.0000121868923" "0.0116479330505" "0.0026122879890" 
##                Mg                Mn                Na                Ni 
## "0.0044485124484" "0.0002055384383" "0.0019301914566" "0.0000145341946" 
##                 P                Pb                 S                Sc 
## "0.0008531412455" "0.0000052149965" "0.0008708952211" "0.0000015134396" 
##                Sr                Ti                 V                Zn 
## "0.0001505037318" "0.0001694551055" "0.0000261315210" "0.0000310939456" 
##               Res 
## "0.9411701318082" 
## attr(,"class")
## [1] "acomp"  
  # Print total variation
cat("Total variation")  
  ## Total variation  
  descstats$totvar  
  ## [1] 1.692286  
 
 
 Normality tests 
  # Set the background color to white for the plots
par(bg = "white")
par(mar = c(4, 4, 1, 1))  # Set margins to avoid too large space

# Evaluate multivariate normality on the ilr-transformed dataset using the Energy test
dataset_ilr_mvn <- mvn(
  data = ilr(comp),
  mvn_test = "energy",
  desc = TRUE
)
dataset_ilr_mvn$multivariate_normality  
 
 
 
  # Evaluate univariate normality (of each component) on the clr-transformed dataset using Shapiro-Wilk test
dataset_clr_mvn <- mvn(
  data = clr(comp),
  univariate_test = "SW",
  desc = TRUE
)
dataset_clr_mvn$univariate_normality  
 
 
 
 
 
 Correlation analysis 
  # Calculate compositional correlation (see Kynclova et al. 2017)
Rs <- corCoDa(comp, method = "spearman")
colnames(Rs) <- colnames(comp) 
rownames(Rs) <- colnames(comp)

# Plot correlation matrix
ggcorrplot(Rs,
           method = "square",
           type = "lower",
           lab = TRUE,       # Show correlation values
           lab_size = 3,     # Adjust text size
           outline.color = "black",  # Make circles more visible
           ggtheme = theme_minimal(), 
           title = "Compositional Correlation Matrix")  
   
  # Calculate non-compositional correlation 
Rs2 <- cor(log10(comp), method = "spearman")
colnames(Rs2) <- colnames(comp) 
rownames(Rs2) <- colnames(comp)

# Plot correlation matrix
ggcorrplot(Rs2,
           method = "square",
           type = "lower",
           lab = TRUE,
           lab_size = 4,
           outline.color = "black",
           ggtheme = theme_minimal(),
           title = "A) Non-Compositional Correlation Matrix")  
   
 
 
 Principal Components Analysis 
  source("./source/quick_pca_screeplot.R")
source("./source/quick_pca_biplot.R")

# Calculating PCA after clr transformation
pca_result <- prcomp(clr(comp), .stand = TRUE)
quick_pca_screeplot(pca_result)  
   
  quick_pca_biplot(pca_result, x = "PC1", y = "PC2", 
                 arrow_scale_factor = "auto", 
                 add_labels = FALSE)  
   
 
 
 Analysis of variance (by groups) 
  # Analysis by "Area"
## Check dispersion homogeneity
aitch_dist <- dist(ilr(comp))
betadisper_results <- betadisper(aitch_dist, Area)
permutest(betadisper_results, pairwise = FALSE) # Check for homogeneity of dispersion  
  ## 
## Permutation test for homogeneity of multivariate dispersions
## Permutation: free
## Number of permutations: 999
## 
## Response: Distances
##           Df  Sum Sq Mean Sq     F N.Perm Pr(>F)
## Groups     1  0.4857 0.48567 1.884    999  0.156
## Residuals 68 17.5295 0.25779  
  boxplot(betadisper_results, main = "Group Dispersions")  
   
  plot(betadisper_results)  
   
  ## Perform perMANOVA
adonis2(aitch_dist ~ Area, permutations = 999)  
 
 
 
  # Analysis by "Activity"
## Check dispersion homogeneity
aitch_dist <- dist(ilr(comp))
betadisper_results <- betadisper(aitch_dist, Activity)
permutest(betadisper_results, pairwise = FALSE) # Check for homogeneity of dispersion  
  ## 
## Permutation test for homogeneity of multivariate dispersions
## Permutation: free
## Number of permutations: 999
## 
## Response: Distances
##           Df  Sum Sq Mean Sq      F N.Perm Pr(>F)   
## Groups     1  2.0144  2.0144 9.0944    999  0.005 **
## Residuals 68 15.0621  0.2215                        
## ---
## Signif. codes:  0 '***' 0.001 '**' 0.01 '*' 0.05 '.' 0.1 ' ' 1  
  boxplot(betadisper_results, main = "Group Dispersions")  
   
  plot(betadisper_results)  
   
  ## Perform perMANOVA
adonis2(aitch_dist ~ Activity, permutations = 999)  
 
 
 
 
 
 Spatial analysis 
  # Create spatial points data frame with clr coords.
spdf_clr <- SpatialPointsDataFrame(coords = coords, data = clr(comp)) 

# Create elemental maps from CLR data
pairsmap(data = spdf_clr@data[, sort(colnames(spdf_clr@data))], loc = spdf_clr@coords)  
   
  # Perform cokriging
## Load requiered functions
source("./source/lag_distance_from_spdf.R")
source("./source/site_diagonal_from_spdf.R")
source("./source/create_gstat_from_spdf.R")
source("./source/calculate_variogram_models.R")
source("./source/fit_lmc_GV.R")
source("./source/create_grid_from_spdf.R")
source("./source/create_ck_maplist.R")
source("./source/ck_cross_validation.R")
source("./source/ck_spatial_validation.R")

## Create and fit variograms
### Create spatial points data frame with olr coords
spdf_ilr <- SpatialPointsDataFrame(coords = coords, data = ilr(comp))
### Calculate the lag distance for variogram width
lag_dist <- lag_distance_from_spdf(spdf_ilr)
### Calculate site diagonal for variogram cutoff
site_diag <- site_diagonal_from_spdf(spdf_ilr)
### Create gstat object using ILR-transformed data
g <- create_gstat_from_spdf(spdf_ilr, method = "ordinary")
### Compute omnidirectional variograms (with cross-variograms)
v <- variogram(g, width = lag_dist / 2, cutoff = site_diag / 3, cross = TRUE)
### Calculate variogram models
g <- calculate_variogram_models(spdf = spdf_ilr, gstat = g, variogram = v,
                                vgm_models = c("Sph", "Exp", "Gau"), # Models to fit. vgm() returns a list of available models to be fitted.
                                threshold = 0) # Percentage threshold for model filtering  
  ## Selected models:
##          psill     range kappa   n
## Nug 0.02187528  0.000000     0 300
## Exp 0.04790359 11.766079     0 173
## Gau 0.05876806  1.778628     0  73
## Sph 0.07356800  2.093504     0  54  
  ### Fit Linear Model of Corregionalisation (LMC)
fitted_lmc <- fit_lmc_GV(v, g$model)
### Plot variogram with LMC
plot(v, fitted_lmc, main = "Fitted variogram")  
   
  ## Produce cokriging maps
### Create new gstat object with LMC for cokriging
g <- create_gstat_from_spdf(spdf_ilr, method = "ordinary")
g$model <- fitted_lmc
### Create grid
grid <- create_grid_from_spdf(spdf_ilr, resolution = 0.25, buffer = 1.8, convex_hull = TRUE)
### Perform cokriging
ck <- predict(g, newdata = grid)  
  ## Linear Model of Coregionalization found. Good.
## [using ordinary cokriging]  
  ### Perform k-fold cross-validation to assess predictive performance
ck_cross_validation(spdf_ilr, g, nfold = 5, plot_scatter = TRUE, plot_hist = TRUE, plot_qq = TRUE)  
  ##                V1            V2            V3            V4            V5
## ME  -0.0097154096  0.0038199772  0.0045399699 -0.0073530076  0.0006593580
## MSE   0.154152799   0.094460153   0.069511565   0.030038353   0.118383363
##                V6            V7            V8            V9           V10
## ME  -0.0011627847 -0.0071100170 -0.0064867776 -0.0057584717 -0.0062329564
## MSE   0.061389700   0.042182742   0.021908964   0.019028254   0.016819995
##               V11           V12           V13           V14           V15
## ME  -0.0001900364 -0.0017036300 -0.0044793186  0.0180213143 -0.0052044271
## MSE   0.034569643   0.005453684   0.013472920   0.113237790   0.016218209
##               V16           V17           V18           V19           V20
## ME  -0.0070978382 -0.0058731728  0.0028844228 -0.0027898897 -0.0033255923
## MSE   0.048008177   0.037048931   0.064454801   0.080932135   0.062110861
##               V21           V22           V23           V24
## ME  -0.0088231277 -0.0040752085 -0.0008945773 -0.0025456170
## MSE   0.051594816   0.019142802   0.022156900   0.022119606  
     
  ### Evaluate spatial prediction accuracy over the interpolation grid
ck_spatial_validation(spdf_ilr, ck, accuracy_plot = TRUE)  
   
  ## $mv_results
##   Accuracy Precision Goodness
## 1     0.95      0.45     0.73
## 
## $var_results
##    Variable Accuracy Precision Goodness
## 1        V1     0.90      0.88     0.94
## 2        V2     0.65      0.95     0.97
## 3        V3     0.80      0.86     0.92
## 4        V4     0.95      0.87     0.94
## 5        V5     0.95      0.85     0.92
## 6        V6     0.90      0.83     0.91
## 7        V7     0.90      0.94     0.97
## 8        V8     0.70      0.95     0.97
## 9        V9     0.85      0.92     0.96
## 10      V10     0.90      0.88     0.94
## 11      V11     0.95      0.88     0.94
## 12      V12     0.80      0.87     0.93
## 13      V13     0.90      0.88     0.94
## 14      V14     0.85      0.91     0.95
## 15      V15     0.35      0.97     0.93
## 16      V16     0.90      0.88     0.94
## 17      V17     0.95      0.86     0.93
## 18      V18     0.85      0.92     0.96
## 19      V19     0.90      0.89     0.94
## 20      V20     0.90      0.84     0.92
## 21      V21     0.70      0.91     0.95
## 22      V22     0.90      0.88     0.94
## 23      V23     0.65      0.96     0.97
## 24      V24     0.90      0.85     0.92  
  ### Plot maps arranged in a grid
ck_maplist <- create_ck_maplist(ck, orig = comp, 
                                compositional_transformation = "ilr", 
                                shapefile = shp)
ck_maplist_m <- lapply(ck_maplist, function(p) {
  p +
    theme_void(base_size = 12) +  # limpia todo salvo texto útil
    theme(
      plot.title = element_text(size = 14, face = "bold", hjust = 0.5),
      legend.title = element_text(size = 10),
      legend.text = element_text(size = 8),
      plot.margin = margin(3, 3, 3, 3)
    ) 
})
grid.arrange(grobs = ck_maplist_m[1:12], ncol = 4, top = "Compositional Ordinary Cokriging")  
   
  grid.arrange(grobs = ck_maplist_m[13:24], ncol = 4, top = "Compositional Ordinary Cokriging")  
   
  ## Explore log-ratio balance maps 
### Hierarchical clustering on the variation matrix
dd <- as.dist(descstats$variation)
hc <- hclust(dd, method = "ward.D")
plot(hc)  
   
  ### Create sequential binary partition (SBP)
vars <- c("Ag","Al","As","B","Ba","Bi","Ca","Co","Cr","Cu","Fe","K","Mg","Mn",
          "Na","Ni","P","Pb","S","Sc","Sr","Ti","V","Zn","Res")
Vsigns <- matrix(0L, nrow = length(vars) - 1, ncol = length(vars),
                 dimnames = list(NULL, vars))
idx <- function(x) match(x, vars) # Helper function for indexes

Vsigns[1,  idx(setdiff(vars, "Res"))] <-  1
Vsigns[1,  idx("Res")]                <- -1
Vsigns[2, idx(c("Ca","Sr","Sc","Zn","Cu","Mg","Ba","Ti","Pb","V","Mn","Ni","Cr","Fe","Al","Co"))] <- -1
Vsigns[2, idx(c("Ag","As","Bi","Na","B","K","P","S"))] <- 1
Vsigns[3, idx("Ag")] <- 1
Vsigns[3, idx(c("As","Bi","Na","B","K","P","S"))] <- -1
Vsigns[4, idx(c("As","Bi"))] <- 1
Vsigns[4, idx(c("Na","B","K","P","S"))] <- -1
Vsigns[5, idx("Na")] <- 1
Vsigns[5, idx(c("B","K","P","S"))] <- -1
Vsigns[6, idx("B")] <- 1
Vsigns[6, idx(c("K","P","S"))] <- -1
Vsigns[7, idx("K")] <- 1
Vsigns[7, idx(c("P","S"))] <- -1
Vsigns[8, idx("P")] <- 1
Vsigns[8, idx("S")] <- -1
Vsigns[9, idx("As")] <- 1
Vsigns[9, idx("Bi")] <- -1
Vsigns[10, idx(c("Ca","Sr"))] <- 1
Vsigns[10, idx(c("Sc","Zn","Cu","Mg","Ba","Ti","Pb","V","Mn","Ni","Cr","Fe","Al","Co"))] <- -1
Vsigns[11, idx("Ca")] <-  1
Vsigns[11, idx("Sr")] <- -1
Vsigns[12, idx("Sc")] <-  1
Vsigns[12, idx(c("Zn","Cu","Mg","Ba","Ti","Pb","V","Mn","Ni","Cr","Fe","Al","Co"))] <- -1
Vsigns[13, idx(c("Zn","Cu","Mg","Ba"))] <-  1
Vsigns[13, idx(c("Ti","Pb","V","Mn","Ni","Cr","Fe","Al","Co"))] <- -1
Vsigns[14, idx(c("Zn","Cu","Mg"))] <-  1
Vsigns[14, idx("Ba")] <- -1
Vsigns[15, idx("Zn")] <- 1
Vsigns[15, idx(c("Cu","Mg"))] <- -1
Vsigns[16, idx("Cu")] <- 1
Vsigns[16, idx("Mg")] <- -1
Vsigns[17, idx(c("Ti","Pb","Al","Co"))] <-  1
Vsigns[17, idx(c("V","Mn","Ni","Cr","Fe"))] <- -1
Vsigns[18, idx(c("Ti","Pb"))] <-  1
Vsigns[18, idx(c("Al","Co"))] <- -1
Vsigns[19, idx("Ti")] <-  1
Vsigns[19, idx("Pb")] <- -1
Vsigns[20, idx("Al")] <-  1
Vsigns[20, idx("Co")] <- -1
Vsigns[21, idx("V")] <- 1
Vsigns[21, idx(c("Mn","Ni","Cr","Fe"))] <- -1
Vsigns[22, idx("Fe")] <- 1
Vsigns[22, idx(c("Mn","Ni","Cr"))] <- -1
Vsigns[23, idx("Mn")] <- 1
Vsigns[23, idx(c("Ni","Cr"))] <- -1
Vsigns[24, idx("Ni")] <- 1
Vsigns[24, idx("Cr")] <- -1

### Balance names
balance_names <- c(
  "All / Res",
  "Ag+As+B+Bi+K+Na+P+S / Al+Ba+Ca+Co+Cr+Cu+Fe+Mg+Mn+Ni+Pb+Sc+Sr+Ti+V+Zn",
  "Ag / As+B+Bi+K+Na+P+S",
  "As+Bi / B+K+Na+P+S",
  "Na / B+K+P+S",
  "B / K+P+S",
  "K / P+S",
  "P / S",
  "As / Bi",
  "Ca+Sr / Al+Ba+Co+Cr+Cu+Fe+Mg+Mn+Ni+Pb+Sc+Ti+V+Zn",
  "Ca / Sr",
  "Sc / Al+Ba+Co+Cr+Cu+Fe+Mg+Mn+Ni+Pb+Ti+V+Zn",
  "Ba+Cu+Mg+Zn / Al+Co+Cr+Fe+Mn+Ni+Pb+Ti+V",
  "Cu+Mg+Zn / Ba",
  "Zn / Cu+Mg",
  "Cu / Mg",
  "Al+Co+Pb+Ti / Cr+Fe+Mn+Ni+V",
  "Pb+Ti / Al+Co",
  "Ti / Pb",
  "Al / Co",
  "V / Cr+Fe+Mn+Ni",
  "Fe / Cr+Mn+Ni",
  "Mn / Cr+Ni",
  "Ni / Cr"
)

### Show SBP
print(Vsigns)  
  ##       Ag Al As  B Ba Bi Ca Co Cr Cu Fe  K Mg Mn Na Ni  P Pb  S Sc Sr Ti  V Zn
##  [1,]  1  1  1  1  1  1  1  1  1  1  1  1  1  1  1  1  1  1  1  1  1  1  1  1
##  [2,]  1 -1  1  1 -1  1 -1 -1 -1 -1 -1  1 -1 -1  1 -1  1 -1  1 -1 -1 -1 -1 -1
##  [3,]  1  0 -1 -1  0 -1  0  0  0  0  0 -1  0  0 -1  0 -1  0 -1  0  0  0  0  0
##  [4,]  0  0  1 -1  0  1  0  0  0  0  0 -1  0  0 -1  0 -1  0 -1  0  0  0  0  0
##  [5,]  0  0  0 -1  0  0  0  0  0  0  0 -1  0  0  1  0 -1  0 -1  0  0  0  0  0
##  [6,]  0  0  0  1  0  0  0  0  0  0  0 -1  0  0  0  0 -1  0 -1  0  0  0  0  0
##  [7,]  0  0  0  0  0  0  0  0  0  0  0  1  0  0  0  0 -1  0 -1  0  0  0  0  0
##  [8,]  0  0  0  0  0  0  0  0  0  0  0  0  0  0  0  0  1  0 -1  0  0  0  0  0
##  [9,]  0  0  1  0  0 -1  0  0  0  0  0  0  0  0  0  0  0  0  0  0  0  0  0  0
## [10,]  0 -1  0  0 -1  0  1 -1 -1 -1 -1  0 -1 -1  0 -1  0 -1  0 -1  1 -1 -1 -1
## [11,]  0  0  0  0  0  0  1  0  0  0  0  0  0  0  0  0  0  0  0  0 -1  0  0  0
## [12,]  0 -1  0  0 -1  0  0 -1 -1 -1 -1  0 -1 -1  0 -1  0 -1  0  1  0 -1 -1 -1
## [13,]  0 -1  0  0  1  0  0 -1 -1  1 -1  0  1 -1  0 -1  0 -1  0  0  0 -1 -1  1
## [14,]  0  0  0  0 -1  0  0  0  0  1  0  0  1  0  0  0  0  0  0  0  0  0  0  1
## [15,]  0  0  0  0  0  0  0  0  0 -1  0  0 -1  0  0  0  0  0  0  0  0  0  0  1
## [16,]  0  0  0  0  0  0  0  0  0  1  0  0 -1  0  0  0  0  0  0  0  0  0  0  0
## [17,]  0  1  0  0  0  0  0  1 -1  0 -1  0  0 -1  0 -1  0  1  0  0  0  1 -1  0
## [18,]  0 -1  0  0  0  0  0 -1  0  0  0  0  0  0  0  0  0  1  0  0  0  1  0  0
## [19,]  0  0  0  0  0  0  0  0  0  0  0  0  0  0  0  0  0 -1  0  0  0  1  0  0
## [20,]  0  1  0  0  0  0  0 -1  0  0  0  0  0  0  0  0  0  0  0  0  0  0  0  0
## [21,]  0  0  0  0  0  0  0  0 -1  0 -1  0  0 -1  0 -1  0  0  0  0  0  0  1  0
## [22,]  0  0  0  0  0  0  0  0 -1  0  1  0  0 -1  0 -1  0  0  0  0  0  0  0  0
## [23,]  0  0  0  0  0  0  0  0 -1  0  0  0  0  1  0 -1  0  0  0  0  0  0  0  0
## [24,]  0  0  0  0  0  0  0  0 -1  0  0  0  0  0  0  1  0  0  0  0  0  0  0  0
##       Res
##  [1,]  -1
##  [2,]   0
##  [3,]   0
##  [4,]   0
##  [5,]   0
##  [6,]   0
##  [7,]   0
##  [8,]   0
##  [9,]   0
## [10,]   0
## [11,]   0
## [12,]   0
## [13,]   0
## [14,]   0
## [15,]   0
## [16,]   0
## [17,]   0
## [18,]   0
## [19,]   0
## [20,]   0
## [21,]   0
## [22,]   0
## [23,]   0
## [24,]   0  
  ### Build ILR basis from SBP and compute balances
V <- gsi.buildilrBase(t(Vsigns))
Xbalances <- ilr(comp, V)
### Summary of SBP and balance statistics
sbp_table <- as.data.frame(Vsigns)
rownames(sbp_table) <- paste0("ilr", seq_len(nrow(sbp_table)), "(x)")
bal_mean <- colMeans(Xbalances, na.rm = TRUE) 
bal_var  <- apply(Xbalances, 2, var, na.rm = TRUE)
tot_var <- sum(bal_var)
pct_var <- 100 * bal_var / tot_var
### Combine SBP structure with balance statistics
table_out <- cbind(
  sbp_table,
  Mean = round(bal_mean, 2),
  Variance = round(bal_var, 2),
  `% Variance` = round(pct_var, 2)
)
table_out  
 
 
 
  ### Perform cokriging with log-ratio balances
spdf_ilr  <- SpatialPointsDataFrame(coords = coords, data = Xbalances)
lag_dist  <- lag_distance_from_spdf(spdf_ilr)
site_diag <- site_diagonal_from_spdf(spdf_ilr)
grid      <- create_grid_from_spdf(spdf_ilr, resolution = 0.25, buffer = 1.8, convex_hull = TRUE)
g <- create_gstat_from_spdf(spdf_ilr, method = "ordinary")
v <- variogram(g, width = lag_dist / 2, cutoff = site_diag / 3, cross = TRUE)
g <- calculate_variogram_models(
  spdf       = spdf_ilr,
  gstat      = g,
  variogram  = v,
  vgm_models = c("Sph", "Exp", "Gau"),  # candidate models to fit
  threshold  = 0                         # percentage threshold for model filtering
)  
  ## Selected models:
##          psill     range kappa   n
## Nug 0.02376065  0.000000     0 300
## Exp 0.05390609 10.866657     0 176
## Sph 0.06304063  1.017887     0  70
## Gau 0.03180271  1.995515     0  54  
  fitted_lmc <- fit_lmc_GV(v, g$model)
g <- create_gstat_from_spdf(spdf_ilr, method = "ordinary")
g$model <- fitted_lmc
ck <- predict(g, newdata = grid)  
  ## Linear Model of Coregionalization found. Good.
## [using ordinary cokriging]  
  ### Cokriging performance
ck_cross_validation(spdf_ilr, g, nfold = 5, plot_scatter = TRUE, plot_hist = TRUE, plot_qq = TRUE)  
  ##                  V1              V2              V3              V4
## ME  -0.000096968321  0.010889570337  0.006060147076 -0.005030306009
## MSE     0.020516199     0.272375307     0.282295222     0.166015622
##                  V5              V6              V7              V8
## ME  -0.001731325087 -0.005494706938 -0.002339488779 -0.000187418014
## MSE     0.091614653     0.036929905     0.028014933     0.024201792
##                  V9             V10             V11             V12
## ME  -0.005418265021  0.000956462942  0.002011509484 -0.014198910766
## MSE     0.072961548     0.148088650     0.038236958     0.067080105
##                 V13             V14             V15             V16
## ME   0.003434235752 -0.000868635707  0.001508089139  0.000156524215
## MSE     0.036223719     0.019150794     0.012386420     0.006976054
##                 V17             V18             V19             V20
## ME  -0.005110652013 -0.002152769133 -0.001793394550 -0.001347442789
## MSE     0.012606915     0.016730942     0.022300583     0.007096617
##                 V21             V22             V23             V24
## ME   0.000258326239  0.000002838025  0.000386837540  0.000359037267
## MSE     0.007528180     0.002303939     0.005225179     0.002206120  
     
  ck_spatial_validation(spdf_ilr, ck, accuracy_plot = TRUE)  
   
  ## $mv_results
##   Accuracy Precision Goodness
## 1     0.95      0.27     0.64
## 
## $var_results
##    Variable Accuracy Precision Goodness
## 1        V1     0.95      0.86     0.93
## 2        V2     0.70      0.87     0.92
## 3        V3     0.90      0.75     0.87
## 4        V4     0.95      0.78     0.89
## 5        V5     0.95      0.85     0.92
## 6        V6     0.40      0.97     0.95
## 7        V7     0.95      0.80     0.90
## 8        V8     0.85      0.83     0.92
## 9        V9     0.80      0.82     0.90
## 10      V10     0.90      0.76     0.88
## 11      V11     0.90      0.82     0.91
## 12      V12     0.95      0.80     0.90
## 13      V13     0.75      0.88     0.93
## 14      V14     0.55      0.94     0.95
## 15      V15     0.90      0.82     0.91
## 16      V16     0.75      0.88     0.94
## 17      V17     0.80      0.88     0.94
## 18      V18     0.95      0.85     0.92
## 19      V19     0.60      0.95     0.94
## 20      V20     0.85      0.88     0.93
## 21      V21     0.95      0.82     0.91
## 22      V22     0.85      0.92     0.96
## 23      V23     0.80      0.93     0.96
## 24      V24     0.90      0.90     0.95  
  ### Map creation (balances)
ck_maplist <- create_ck_maplist(
  ck,
  orig= comp,
  compositional_transformation = "none",
  shapefile = shp
)

ck_maplist_m <- lapply(ck_maplist, function(p) {
  p +
    theme_void(base_size = 12) +
    theme(
      plot.title   = element_text(size = 12, face = "bold", hjust = 0.5),
      legend.title = element_text(size = 10),
      legend.text  = element_text(size = 8),
      plot.margin  = margin(3, 3, 3, 3)
    )
})

balance_names_wrapped <- balance_names |>
  str_replace_all(" / ", " /\n") |>
  str_wrap(width = 20)
for (i in seq_along(ck_maplist_m)) {
  ck_maplist_m[[i]] <- ck_maplist_m[[i]] + ggtitle(balance_names_wrapped[i])
}
names(ck_maplist_m) <- balance_names_wrapped

grid.arrange(grobs = ck_maplist_m[1:12],  ncol = 4, top = "Compositional Ordinary Cokriging (Balances)")  
   
  grid.arrange(grobs = ck_maplist_m[13:24], ncol = 4, top = "Compositional Ordinary Cokriging (Balances)")  
   
  ### Uncertainty maps for log-ratio balances

# Prediction and variance columns from cokriging output
pred_cols <- grep("\\.pred$", names(ck), value = TRUE)
var_cols  <- grep("\\.var$",  names(ck), value = TRUE)

# Add standard error columns if they do not exist
for (v in var_cols) {
  se_name <- sub("\\.var$", ".se", v)
  if (!se_name %in% names(ck)) {
    ck[[se_name]] <- sqrt(ck[[v]])
  }
}

# Convert cokriging result to data frame
ck_df <- as.data.frame(ck)

# Detect coordinate names
xcol <- if ("x" %in% names(ck_df)) "x" else if ("coords.x1" %in% names(ck_df)) "coords.x1" else names(ck_df)[1]
ycol <- if ("y" %in% names(ck_df)) "y" else if ("coords.x2" %in% names(ck_df)) "coords.x2" else names(ck_df)[2]

# Convert site outline to plain coordinates
outline_sf <- st_set_crs(shp, NA)
outline_coords <- st_coordinates(outline_sf)
outline_df <- as.data.frame(outline_coords)

# Common map extents
x_rng <- range(ck_df[[xcol]], na.rm = TRUE)
y_rng <- range(ck_df[[ycol]], na.rm = TRUE)

# Wrap balance names for plot titles
balance_names_wrapped <- balance_names |>
  str_replace_all(" / ", " /\n") |>
  str_wrap(width = 20)

# Create uncertainty maps
balance_uncertainty_maps <- vector("list", length(pred_cols))

for (i in seq_along(pred_cols)) {
  
  se_col <- sub("\\.pred$", ".se", pred_cols[i])
  
  balance_uncertainty_maps[[i]] <- ggplot() +
    geom_raster(
      data = ck_df,
      aes(x = .data[[xcol]], y = .data[[ycol]], fill = .data[[se_col]])
    ) +
    geom_path(
      data = outline_df,
      aes(X, Y, group = L1),
      colour = "black",
      linewidth = 0.45
    ) +
    coord_equal(
      xlim = x_rng,
      ylim = y_rng,
      expand = FALSE
    ) +
    scale_fill_viridis_c(
      option = "magma",
      name = "SE",
      breaks = scales::pretty_breaks(n = 3),
      guide = guide_colorbar(
        title.position = "top",
        title.vjust = 1,
        barwidth = unit(0.8, "cm"),
        barheight = unit(2.8, "cm")
      )
    ) +
    ggtitle(balance_names_wrapped[i]) +
    theme_void(base_size = 12) +
    theme(
      plot.title = element_text(size = 12, face = "bold", hjust = 0.5),
      legend.position = "right",
      legend.direction = "vertical",
      legend.title = element_text(size = 10),
      legend.text = element_text(size = 8),
      plot.margin = margin(3, 3, 3, 3)
    )
}

names(balance_uncertainty_maps) <- balance_names_wrapped

# Plot uncertainty maps in the same layout as prediction maps
grid.arrange(
  grobs = balance_uncertainty_maps[1:12],
  ncol = 4,
  top = "Cokriging Uncertainty (Balances)"
)  
   
  grid.arrange(
  grobs = balance_uncertainty_maps[13:24],
  ncol = 4,
  top = "Cokriging Uncertainty (Balances)"
)  
   
 
 
 PACKAGE REFERENCES 
  pkgs <- c("stats", "utils", "dplyr", "tidyr", "tidyverse", "magrittr", "stringr",
          "sf", "sp", "raster", "rasterVis", "stars", "scales", "gstat", "spdep", "automap",
          "dbscan", "grid", "gridExtra", "lattice", "latticeExtra", "ggplot2",
          "ggdendro", "ggrepel", "ggcorrplot", "ggpubr", "colorspace", "viridis",
          "patchwork", "leaflet", "classInt", "knitr", "kableExtra", "readxl",
          "jsonlite", "htmlwidgets", "IRdisplay", "car", "dendextend", "MASS",
          "MVN", "RVAideMemoire", "vegan", "NbClust", "biotools", "rrcov",
          "DescTools", "compositions", "zCompositions", "robCompositions",
          "gmGeostats")

citas <- lapply(pkgs, citation)

for (p in pkgs) {
  cat("\n\n====================\n")
  cat("PACKAGE:", p, "\n")
  cat("====================\n")
  print(citation(p))
}  
  ## 
## 
## ====================
## PACKAGE: stats 
## ====================
## The 'stats' package is part of R.  To cite R in publications use:
## 
##   R Core Team (2024). _R: A Language and Environment for Statistical
##   Computing_. R Foundation for Statistical Computing, Vienna, Austria.
##   <https://www.R-project.org/>.
## 
## A BibTeX entry for LaTeX users is
## 
##   @Manual{,
##     title = {R: A Language and Environment for Statistical Computing},
##     author = {{R Core Team}},
##     organization = {R Foundation for Statistical Computing},
##     address = {Vienna, Austria},
##     year = {2024},
##     url = {https://www.R-project.org/},
##   }
## 
## We have invested a lot of time and effort in creating R, please cite it
## when using it for data analysis. See also 'citation("pkgname")' for
## citing R packages.
## 
## 
## ====================
## PACKAGE: utils 
## ====================
## The 'utils' package is part of R.  To cite R in publications use:
## 
##   R Core Team (2024). _R: A Language and Environment for Statistical
##   Computing_. R Foundation for Statistical Computing, Vienna, Austria.
##   <https://www.R-project.org/>.
## 
## A BibTeX entry for LaTeX users is
## 
##   @Manual{,
##     title = {R: A Language and Environment for Statistical Computing},
##     author = {{R Core Team}},
##     organization = {R Foundation for Statistical Computing},
##     address = {Vienna, Austria},
##     year = {2024},
##     url = {https://www.R-project.org/},
##   }
## 
## We have invested a lot of time and effort in creating R, please cite it
## when using it for data analysis. See also 'citation("pkgname")' for
## citing R packages.
## 
## 
## ====================
## PACKAGE: dplyr 
## ====================
## To cite package 'dplyr' in publications use:
## 
##   Wickham H, François R, Henry L, Müller K, Vaughan D (2023). _dplyr: A
##   Grammar of Data Manipulation_. R package version 1.1.4,
##   <https://CRAN.R-project.org/package=dplyr>.
## 
## A BibTeX entry for LaTeX users is
## 
##   @Manual{,
##     title = {dplyr: A Grammar of Data Manipulation},
##     author = {Hadley Wickham and Romain François and Lionel Henry and Kirill Müller and Davis Vaughan},
##     year = {2023},
##     note = {R package version 1.1.4},
##     url = {https://CRAN.R-project.org/package=dplyr},
##   }
## 
## 
## ====================
## PACKAGE: tidyr 
## ====================
## To cite package 'tidyr' in publications use:
## 
##   Wickham H, Vaughan D, Girlich M (2024). _tidyr: Tidy Messy Data_. R
##   package version 1.3.1, <https://CRAN.R-project.org/package=tidyr>.
## 
## A BibTeX entry for LaTeX users is
## 
##   @Manual{,
##     title = {tidyr: Tidy Messy Data},
##     author = {Hadley Wickham and Davis Vaughan and Maximilian Girlich},
##     year = {2024},
##     note = {R package version 1.3.1},
##     url = {https://CRAN.R-project.org/package=tidyr},
##   }
## 
## 
## ====================
## PACKAGE: tidyverse 
## ====================
## To cite package 'tidyverse' in publications use:
## 
##   Wickham H, Averick M, Bryan J, Chang W, McGowan LD, François R,
##   Grolemund G, Hayes A, Henry L, Hester J, Kuhn M, Pedersen TL, Miller
##   E, Bache SM, Müller K, Ooms J, Robinson D, Seidel DP, Spinu V,
##   Takahashi K, Vaughan D, Wilke C, Woo K, Yutani H (2019). "Welcome to
##   the tidyverse." _Journal of Open Source Software_, *4*(43), 1686.
##   doi:10.21105/joss.01686 <https://doi.org/10.21105/joss.01686>.
## 
## A BibTeX entry for LaTeX users is
## 
##   @Article{,
##     title = {Welcome to the {tidyverse}},
##     author = {Hadley Wickham and Mara Averick and Jennifer Bryan and Winston Chang and Lucy D'Agostino McGowan and Romain François and Garrett Grolemund and Alex Hayes and Lionel Henry and Jim Hester and Max Kuhn and Thomas Lin Pedersen and Evan Miller and Stephan Milton Bache and Kirill Müller and Jeroen Ooms and David Robinson and Dana Paige Seidel and Vitalie Spinu and Kohske Takahashi and Davis Vaughan and Claus Wilke and Kara Woo and Hiroaki Yutani},
##     year = {2019},
##     journal = {Journal of Open Source Software},
##     volume = {4},
##     number = {43},
##     pages = {1686},
##     doi = {10.21105/joss.01686},
##   }
## 
## 
## ====================
## PACKAGE: magrittr 
## ====================
## To cite package 'magrittr' in publications use:
## 
##   Bache S, Wickham H (2022). _magrittr: A Forward-Pipe Operator for R_.
##   R package version 2.0.3, https://github.com/tidyverse/magrittr,
##   <https://magrittr.tidyverse.org>.
## 
## A BibTeX entry for LaTeX users is
## 
##   @Manual{,
##     title = {magrittr: A Forward-Pipe Operator for R},
##     author = {Stefan Milton Bache and Hadley Wickham},
##     year = {2022},
##     note = {R package version 2.0.3, 
## https://github.com/tidyverse/magrittr},
##     url = {https://magrittr.tidyverse.org},
##   }
## 
## 
## ====================
## PACKAGE: stringr 
## ====================
## To cite package 'stringr' in publications use:
## 
##   Wickham H (2023). _stringr: Simple, Consistent Wrappers for Common
##   String Operations_. R package version 1.5.1,
##   <https://CRAN.R-project.org/package=stringr>.
## 
## A BibTeX entry for LaTeX users is
## 
##   @Manual{,
##     title = {stringr: Simple, Consistent Wrappers for Common String Operations},
##     author = {Hadley Wickham},
##     year = {2023},
##     note = {R package version 1.5.1},
##     url = {https://CRAN.R-project.org/package=stringr},
##   }
## 
## 
## ====================
## PACKAGE: sf 
## ====================
## To cite package sf in publications, please use:
## 
##   Pebesma, E., & Bivand, R. (2023). Spatial Data Science: With
##   Applications in R. Chapman and Hall/CRC.
##   https://doi.org/10.1201/9780429459016
## 
##   Pebesma, E., 2018. Simple Features for R: Standardized Support for
##   Spatial Vector Data. The R Journal 10 (1), 439-446,
##   https://doi.org/10.32614/RJ-2018-009
## 
## To see these entries in BibTeX format, use 'print(<citation>,
## bibtex=TRUE)', 'toBibtex(.)', or set
## 'options(citation.bibtex.max=999)'.
## 
## 
## ====================
## PACKAGE: sp 
## ====================
## To cite package sp in publications use:
## 
##   Pebesma E, Bivand R (2005). "Classes and methods for spatial data in
##   R." _R News_, *5*(2), 9-13. <https://CRAN.R-project.org/doc/Rnews/>.
## 
##   Bivand R, Pebesma E, Gomez-Rubio V (2013). _Applied spatial data
##   analysis with R, Second edition_. Springer, NY.
##   <https://asdar-book.org/>.
## 
## To see these entries in BibTeX format, use 'print(<citation>,
## bibtex=TRUE)', 'toBibtex(.)', or set
## 'options(citation.bibtex.max=999)'.
## 
## 
## ====================
## PACKAGE: raster 
## ====================
## To cite package 'raster' in publications use:
## 
##   Hijmans R (2025). _raster: Geographic Data Analysis and Modeling_. R
##   package version 3.6-31, <https://CRAN.R-project.org/package=raster>.
## 
## A BibTeX entry for LaTeX users is
## 
##   @Manual{,
##     title = {raster: Geographic Data Analysis and Modeling},
##     author = {Robert J. Hijmans},
##     year = {2025},
##     note = {R package version 3.6-31},
##     url = {https://CRAN.R-project.org/package=raster},
##   }
## 
## 
## ====================
## PACKAGE: rasterVis 
## ====================
## To cite package 'rasterVis' in publications use:
## 
##   Oscar Perpinan Lamigueiro and Robert Hijmans (2023), rasterVis. R
##   package version 0.51.6.
## 
## A BibTeX entry for LaTeX users is
## 
##   @Manual{,
##     title = {{rasterVis}},
##     author = {{Oscar Perpi{\~n}{\'a}n} and {Robert Hijmans}},
##     year = {2023},
##     url = {https://oscarperpinan.github.io/rastervis/},
##     note = {R package version 0.51.6},
##   }
## 
## 
## ====================
## PACKAGE: stars 
## ====================
## To cite package stars in publications, please use:
## 
##   Pebesma E, Bivand R (2023). _Spatial Data Science: With applications
##   in R_. Chapman and Hall/CRC, London. doi:10.1201/9780429459016
##   <https://doi.org/10.1201/9780429459016>,
##   <https://r-spatial.org/book/>.
## 
## A BibTeX entry for LaTeX users is
## 
##   @Book{,
##     title = {{Spatial Data Science: With applications in R}},
##     author = {Edzer Pebesma and Roger Bivand},
##     year = {2023},
##     publisher = {Chapman and Hall/CRC},
##     address = {London},
##     url = {https://r-spatial.org/book/},
##     pages = {352},
##     doi = {10.1201/9780429459016},
##   }
## 
## 
## ====================
## PACKAGE: scales 
## ====================
## To cite package 'scales' in publications use:
## 
##   Wickham H, Pedersen T, Seidel D (2025). _scales: Scale Functions for
##   Visualization_. R package version 1.4.0,
##   <https://CRAN.R-project.org/package=scales>.
## 
## A BibTeX entry for LaTeX users is
## 
##   @Manual{,
##     title = {scales: Scale Functions for Visualization},
##     author = {Hadley Wickham and Thomas Lin Pedersen and Dana Seidel},
##     year = {2025},
##     note = {R package version 1.4.0},
##     url = {https://CRAN.R-project.org/package=scales},
##   }
## 
## 
## ====================
## PACKAGE: gstat 
## ====================
## To cite package gstat in publications use:
## 
##   Pebesma, E.J., 2004. Multivariable geostatistics in S: the gstat
##   package. Computers & Geosciences, 30: 683-691.
## 
##   Benedikt Gräler, Edzer Pebesma and Gerard Heuvelink, 2016.
##   Spatio-Temporal Interpolation using gstat.  The R Journal 8(1),
##   204-218
## 
## To see these entries in BibTeX format, use 'print(<citation>,
## bibtex=TRUE)', 'toBibtex(.)', or set
## 'options(citation.bibtex.max=999)'.
## 
## 
## ====================
## PACKAGE: spdep 
## ====================
## To cite spdep in publications use one or more of the following as
## appropriate:
## 
##   Bivand R, Wong D (2018). "Comparing implementations of global and
##   local indicators of spatial association." _TEST_, *27*(3), 716-748.
##   doi:10.1007/s11749-018-0599-x
##   <https://doi.org/10.1007/s11749-018-0599-x>.
## 
##   Roger Bivand (2022). "R Packages for Analyzing Spatial Data: A
##   Comparative Case Study with Areal Data." _Geographical Analysis_,
##   *54*(3), 488-518. doi:10.1111/gean.12319
##   <https://doi.org/10.1111/gean.12319>.
## 
##   Bivand R, Pebesma E, Gómez-Rubio V (2013). _Applied spatial data
##   analysis with R, Second edition_. Springer, NY.
##   <https://asdar-book.org/>.
## 
##   Pebesma E, Bivand R (2023). _Spatial Data Science With Applications
##   in R_. Chapman & Hall. <https://r-spatial.org/book/>.
## 
## To see these entries in BibTeX format, use 'print(<citation>,
## bibtex=TRUE)', 'toBibtex(.)', or set
## 'options(citation.bibtex.max=999)'.
## 
## 
## ====================
## PACKAGE: automap 
## ====================
## To cite package automap in publications use:
## 
##   Hiemstra, P.H., Pebesma, E.J., Twenhofel, C.J.W. and G.B.M.
##   Heuvelink, 2008. Real-time automatic interpolation of ambient gamma
##   dose rates from the Dutch Radioactivity Monitoring Network. Computers
##   & Geosciences, accepted for publication.
## 
## A BibTeX entry for LaTeX users is
## 
##   @Article{,
##     title = {Real-time automatic interpolation of ambient gamma dose rates from the Dutch Radioactivity Monitoring Network},
##     author = {P.H. Hiemstra and E.J. Pebesma and C.J.W. Twenh"{o}fel and G.B.M. Heuvelink},
##     journal = {Computers & Geosciences},
##     year = {2008},
##     note = {DOI: http://dx.doi.org/10.1016/j.cageo.2008.10.011},
##   }
## 
## 
## ====================
## PACKAGE: dbscan 
## ====================
## Hahsler M, Piekenbrock M (2025). _dbscan: Density-Based Spatial
## Clustering of Applications with Noise (DBSCAN) and Related Algorithms_.
## R package version 1.2.2, <https://CRAN.R-project.org/package=dbscan>.
## 
## To cite dbscan in publications use:
## 
##   Hahsler M, Piekenbrock M, Doran D (2019). "dbscan: Fast Density-Based
##   Clustering with R." _Journal of Statistical Software_, *91*(1), 1-30.
##   doi:10.18637/jss.v091.i01 <https://doi.org/10.18637/jss.v091.i01>.
## 
## To see these entries in BibTeX format, use 'print(<citation>,
## bibtex=TRUE)', 'toBibtex(.)', or set
## 'options(citation.bibtex.max=999)'.
## 
## 
## ====================
## PACKAGE: grid 
## ====================
## The 'grid' package is part of R.  To cite R in publications use:
## 
##   R Core Team (2024). _R: A Language and Environment for Statistical
##   Computing_. R Foundation for Statistical Computing, Vienna, Austria.
##   <https://www.R-project.org/>.
## 
## A BibTeX entry for LaTeX users is
## 
##   @Manual{,
##     title = {R: A Language and Environment for Statistical Computing},
##     author = {{R Core Team}},
##     organization = {R Foundation for Statistical Computing},
##     address = {Vienna, Austria},
##     year = {2024},
##     url = {https://www.R-project.org/},
##   }
## 
## We have invested a lot of time and effort in creating R, please cite it
## when using it for data analysis. See also 'citation("pkgname")' for
## citing R packages.
## 
## 
## ====================
## PACKAGE: gridExtra 
## ====================
## To cite package 'gridExtra' in publications use:
## 
##   Auguie B (2017). _gridExtra: Miscellaneous Functions for "Grid"
##   Graphics_. R package version 2.3,
##   <https://CRAN.R-project.org/package=gridExtra>.
## 
## A BibTeX entry for LaTeX users is
## 
##   @Manual{,
##     title = {gridExtra: Miscellaneous Functions for "Grid" Graphics},
##     author = {Baptiste Auguie},
##     year = {2017},
##     note = {R package version 2.3},
##     url = {https://CRAN.R-project.org/package=gridExtra},
##   }
## 
## 
## ====================
## PACKAGE: lattice 
## ====================
## To cite package 'lattice' in publications use:
## 
##   Sarkar D (2008). _Lattice: Multivariate Data Visualization with R_.
##   Springer, New York. ISBN 978-0-387-75968-5,
##   <http://lmdvr.r-forge.r-project.org>.
## 
## A BibTeX entry for LaTeX users is
## 
##   @Book{,
##     title = {Lattice: Multivariate Data Visualization with R},
##     author = {Deepayan Sarkar},
##     year = {2008},
##     publisher = {Springer},
##     address = {New York},
##     isbn = {978-0-387-75968-5},
##     url = {http://lmdvr.r-forge.r-project.org},
##   }
## 
## 
## ====================
## PACKAGE: latticeExtra 
## ====================
## To cite package 'latticeExtra' in publications use:
## 
##   Sarkar D, Andrews F (2022). _latticeExtra: Extra Graphical Utilities
##   Based on Lattice_. R package version 0.6-30,
##   <https://CRAN.R-project.org/package=latticeExtra>.
## 
## A BibTeX entry for LaTeX users is
## 
##   @Manual{,
##     title = {latticeExtra: Extra Graphical Utilities Based on Lattice},
##     author = {Deepayan Sarkar and Felix Andrews},
##     year = {2022},
##     note = {R package version 0.6-30},
##     url = {https://CRAN.R-project.org/package=latticeExtra},
##   }
## 
## ATTENTION: This citation information has been auto-generated from the
## package DESCRIPTION file and may need manual editing, see
## 'help("citation")'.
## 
## 
## ====================
## PACKAGE: ggplot2 
## ====================
## To cite ggplot2 in publications, please use
## 
##   H. Wickham. ggplot2: Elegant Graphics for Data Analysis.
##   Springer-Verlag New York, 2016.
## 
## A BibTeX entry for LaTeX users is
## 
##   @Book{,
##     author = {Hadley Wickham},
##     title = {ggplot2: Elegant Graphics for Data Analysis},
##     publisher = {Springer-Verlag New York},
##     year = {2016},
##     isbn = {978-3-319-24277-4},
##     url = {https://ggplot2.tidyverse.org},
##   }
## 
## 
## ====================
## PACKAGE: ggdendro 
## ====================
## To cite package 'ggdendro' in publications use:
## 
##   de Vries A, Ripley BD (2024). _ggdendro: Create Dendrograms and Tree
##   Diagrams Using 'ggplot2'_. R package version 0.2.0,
##   <https://CRAN.R-project.org/package=ggdendro>.
## 
## A BibTeX entry for LaTeX users is
## 
##   @Manual{,
##     title = {ggdendro: Create Dendrograms and Tree Diagrams Using 'ggplot2'},
##     author = {Andrie {de Vries} and Brian D. Ripley},
##     year = {2024},
##     note = {R package version 0.2.0},
##     url = {https://CRAN.R-project.org/package=ggdendro},
##   }
## 
## 
## ====================
## PACKAGE: ggrepel 
## ====================
## To cite package 'ggrepel' in publications use:
## 
##   Slowikowski K (2024). _ggrepel: Automatically Position
##   Non-Overlapping Text Labels with 'ggplot2'_. R package version 0.9.6,
##   <https://CRAN.R-project.org/package=ggrepel>.
## 
## A BibTeX entry for LaTeX users is
## 
##   @Manual{,
##     title = {ggrepel: Automatically Position Non-Overlapping Text Labels with
## 'ggplot2'},
##     author = {Kamil Slowikowski},
##     year = {2024},
##     note = {R package version 0.9.6},
##     url = {https://CRAN.R-project.org/package=ggrepel},
##   }
## 
## 
## ====================
## PACKAGE: ggcorrplot 
## ====================
## To cite package 'ggcorrplot' in publications use:
## 
##   Kassambara A (2023). _ggcorrplot: Visualization of a Correlation
##   Matrix using 'ggplot2'_. R package version 0.1.4.1,
##   <https://CRAN.R-project.org/package=ggcorrplot>.
## 
## A BibTeX entry for LaTeX users is
## 
##   @Manual{,
##     title = {ggcorrplot: Visualization of a Correlation Matrix using 'ggplot2'},
##     author = {Alboukadel Kassambara},
##     year = {2023},
##     note = {R package version 0.1.4.1},
##     url = {https://CRAN.R-project.org/package=ggcorrplot},
##   }
## 
## 
## ====================
## PACKAGE: ggpubr 
## ====================
## To cite package 'ggpubr' in publications use:
## 
##   Kassambara A (2023). _ggpubr: 'ggplot2' Based Publication Ready
##   Plots_. R package version 0.6.0,
##   <https://CRAN.R-project.org/package=ggpubr>.
## 
## A BibTeX entry for LaTeX users is
## 
##   @Manual{,
##     title = {ggpubr: 'ggplot2' Based Publication Ready Plots},
##     author = {Alboukadel Kassambara},
##     year = {2023},
##     note = {R package version 0.6.0},
##     url = {https://CRAN.R-project.org/package=ggpubr},
##   }
## 
## 
## ====================
## PACKAGE: colorspace 
## ====================
## To cite colorspace in publications use:
## 
##   Zeileis A, Fisher JC, Hornik K, Ihaka R, McWhite CD, Murrell P,
##   Stauffer R, Wilke CO (2020). "colorspace: A Toolbox for Manipulating
##   and Assessing Colors and Palettes." _Journal of Statistical
##   Software_, *96*(1), 1-49. doi:10.18637/jss.v096.i01
##   <https://doi.org/10.18637/jss.v096.i01>.
## 
## If you use HCL-based color palettes, please cite:
## 
##   Zeileis A, Hornik K, Murrell P (2009). "Escaping RGBland: Selecting
##   Colors for Statistical Graphics." _Computational Statistics & Data
##   Analysis_, *53*(9), 3259-3270. doi:10.1016/j.csda.2008.11.033
##   <https://doi.org/10.1016/j.csda.2008.11.033>.
## 
## If you use HCL-based color palettes in meteorological visualizations,
## please cite:
## 
##   Stauffer R, Mayr GJ, Dabernig M, Zeileis A (2009). "Somewhere over
##   the Rainbow: How to Make Effective Use of Colors in Meteorological
##   Visualizations." _Bulletin of the American Meteorological Society_,
##   *96*(2), 203-216. doi:10.1175/BAMS-D-13-00155.1
##   <https://doi.org/10.1175/BAMS-D-13-00155.1>.
## 
## To see these entries in BibTeX format, use 'print(<citation>,
## bibtex=TRUE)', 'toBibtex(.)', or set
## 'options(citation.bibtex.max=999)'.
## 
## 
## ====================
## PACKAGE: viridis 
## ====================
## To cite viridis/viridisLite in publications use:
## 
##   Simon Garnier, Noam Ross, Robert Rudis, Antônio P. Camargo, Marco
##   Sciaini, and Cédric Scherer (2024). viridis(Lite) -
##   Colorblind-Friendly Color Maps for R. viridis package version 0.6.5.
## 
## A BibTeX entry for LaTeX users is
## 
##   @Manual{,
##     title = {{viridis(Lite)} - Colorblind-Friendly Color Maps for R},
##     author = {{Garnier} and {Simon} and {Ross} and {Noam} and {Rudis} and {Robert} and {Camargo} and Antônio Pedro and {Sciaini} and {Marco} and {Scherer} and {Cédric}},
##     year = {2024},
##     note = {viridis package version 0.6.5},
##     url = {https://sjmgarnier.github.io/viridis/},
##     doi = {10.5281/zenodo.4679423},
##   }
## 
## 
## ====================
## PACKAGE: patchwork 
## ====================
## To cite package 'patchwork' in publications use:
## 
##   Pedersen T (2024). _patchwork: The Composer of Plots_. R package
##   version 1.3.0, <https://CRAN.R-project.org/package=patchwork>.
## 
## A BibTeX entry for LaTeX users is
## 
##   @Manual{,
##     title = {patchwork: The Composer of Plots},
##     author = {Thomas Lin Pedersen},
##     year = {2024},
##     note = {R package version 1.3.0},
##     url = {https://CRAN.R-project.org/package=patchwork},
##   }
## 
## 
## ====================
## PACKAGE: leaflet 
## ====================
## To cite package 'leaflet' in publications use:
## 
##   Cheng J, Schloerke B, Karambelkar B, Xie Y (2024). _leaflet: Create
##   Interactive Web Maps with the JavaScript 'Leaflet' Library_. R
##   package version 2.2.2, <https://CRAN.R-project.org/package=leaflet>.
## 
## A BibTeX entry for LaTeX users is
## 
##   @Manual{,
##     title = {leaflet: Create Interactive Web Maps with the JavaScript 'Leaflet'
## Library},
##     author = {Joe Cheng and Barret Schloerke and Bhaskar Karambelkar and Yihui Xie},
##     year = {2024},
##     note = {R package version 2.2.2},
##     url = {https://CRAN.R-project.org/package=leaflet},
##   }
## 
## 
## ====================
## PACKAGE: classInt 
## ====================
## To cite package 'classInt' in publications use:
## 
##   Bivand R (2025). _classInt: Choose Univariate Class Intervals_. R
##   package version 0.4-11,
##   <https://CRAN.R-project.org/package=classInt>.
## 
## A BibTeX entry for LaTeX users is
## 
##   @Manual{,
##     title = {classInt: Choose Univariate Class Intervals},
##     author = {Roger Bivand},
##     year = {2025},
##     note = {R package version 0.4-11},
##     url = {https://CRAN.R-project.org/package=classInt},
##   }
## 
## 
## ====================
## PACKAGE: knitr 
## ====================
## To cite package 'knitr' in publications use:
## 
##   Xie Y (2024). _knitr: A General-Purpose Package for Dynamic Report
##   Generation in R_. R package version 1.49, <https://yihui.org/knitr/>.
## 
##   Yihui Xie (2015) Dynamic Documents with R and knitr. 2nd edition.
##   Chapman and Hall/CRC. ISBN 978-1498716963
## 
##   Yihui Xie (2014) knitr: A Comprehensive Tool for Reproducible
##   Research in R. In Victoria Stodden, Friedrich Leisch and Roger D.
##   Peng, editors, Implementing Reproducible Computational Research.
##   Chapman and Hall/CRC. ISBN 978-1466561595
## 
## To see these entries in BibTeX format, use 'print(<citation>,
## bibtex=TRUE)', 'toBibtex(.)', or set
## 'options(citation.bibtex.max=999)'.
## 
## 
## ====================
## PACKAGE: kableExtra 
## ====================
## To cite package 'kableExtra' in publications use:
## 
##   Zhu H (2024). _kableExtra: Construct Complex Table with 'kable' and
##   Pipe Syntax_. R package version 1.4.0,
##   <https://CRAN.R-project.org/package=kableExtra>.
## 
## A BibTeX entry for LaTeX users is
## 
##   @Manual{,
##     title = {kableExtra: Construct Complex Table with 'kable' and Pipe Syntax},
##     author = {Hao Zhu},
##     year = {2024},
##     note = {R package version 1.4.0},
##     url = {https://CRAN.R-project.org/package=kableExtra},
##   }
## 
## 
## ====================
## PACKAGE: readxl 
## ====================
## To cite package 'readxl' in publications use:
## 
##   Wickham H, Bryan J (2025). _readxl: Read Excel Files_. R package
##   version 1.4.5, <https://CRAN.R-project.org/package=readxl>.
## 
## A BibTeX entry for LaTeX users is
## 
##   @Manual{,
##     title = {readxl: Read Excel Files},
##     author = {Hadley Wickham and Jennifer Bryan},
##     year = {2025},
##     note = {R package version 1.4.5},
##     url = {https://CRAN.R-project.org/package=readxl},
##   }
## 
## 
## ====================
## PACKAGE: jsonlite 
## ====================
## To cite jsonlite in publications use:
## 
##   Ooms J (2014). "The jsonlite Package: A Practical and Consistent
##   Mapping Between JSON Data and R Objects." _arXiv:1403.2805
##   [stat.CO]_. <https://arxiv.org/abs/1403.2805>.
## 
## A BibTeX entry for LaTeX users is
## 
##   @Article{,
##     title = {The jsonlite Package: A Practical and Consistent Mapping Between JSON Data and R Objects},
##     author = {Jeroen Ooms},
##     journal = {arXiv:1403.2805 [stat.CO]},
##     year = {2014},
##     url = {https://arxiv.org/abs/1403.2805},
##   }
## 
## 
## ====================
## PACKAGE: htmlwidgets 
## ====================
## To cite package 'htmlwidgets' in publications use:
## 
##   Vaidyanathan R, Xie Y, Allaire J, Cheng J, Sievert C, Russell K
##   (2023). _htmlwidgets: HTML Widgets for R_. R package version 1.6.4,
##   <https://CRAN.R-project.org/package=htmlwidgets>.
## 
## A BibTeX entry for LaTeX users is
## 
##   @Manual{,
##     title = {htmlwidgets: HTML Widgets for R},
##     author = {Ramnath Vaidyanathan and Yihui Xie and JJ Allaire and Joe Cheng and Carson Sievert and Kenton Russell},
##     year = {2023},
##     note = {R package version 1.6.4},
##     url = {https://CRAN.R-project.org/package=htmlwidgets},
##   }
## 
## 
## ====================
## PACKAGE: IRdisplay 
## ====================
## To cite package 'IRdisplay' in publications use:
## 
##   Kluyver T, Angerer P, Schulz J (2022). _IRdisplay: 'Jupyter' Display
##   Machinery_. R package version 1.1,
##   <https://CRAN.R-project.org/package=IRdisplay>.
## 
## A BibTeX entry for LaTeX users is
## 
##   @Manual{,
##     title = {IRdisplay: 'Jupyter' Display Machinery},
##     author = {Thomas Kluyver and Philipp Angerer and Jan Schulz},
##     year = {2022},
##     note = {R package version 1.1},
##     url = {https://CRAN.R-project.org/package=IRdisplay},
##   }
## 
## 
## ====================
## PACKAGE: car 
## ====================
## To cite the car package in publications use:
## 
##   Fox J, Weisberg S (2019). _An R Companion to Applied Regression_,
##   Third edition. Sage, Thousand Oaks CA.
##   <https://www.john-fox.ca/Companion/>.
## 
## A BibTeX entry for LaTeX users is
## 
##   @Book{,
##     title = {An {R} Companion to Applied Regression},
##     edition = {Third},
##     author = {John Fox and Sanford Weisberg},
##     year = {2019},
##     publisher = {Sage},
##     address = {Thousand Oaks {CA}},
##     url = {https://www.john-fox.ca/Companion/},
##   }
## 
## 
## ====================
## PACKAGE: dendextend 
## ====================
## The methods within the package can be cited as:
## 
##   Tal Galili (2015). dendextend: an R package for visualizing,
##   adjusting, and comparing trees of hierarchical clustering.
##   Bioinformatics. DOI: 10.1093/bioinformatics/btv428
## 
## A BibTeX entry for LaTeX users is
## 
##   @Article{,
##     author = {Tal Galili},
##     title = {dendextend: an R package for visualizing, adjusting, and comparing trees of hierarchical clustering},
##     journal = {Bioinformatics},
##     year = {2015},
##     doi = {10.1093/bioinformatics/btv428},
##     url = {https://doi.org/10.1093/bioinformatics/btv428},
##     eprint = {https://academic.oup.com/bioinformatics/article-pdf/31/22/3718/17122682/btv428.pdf},
##   }
## 
## This free open-source software implements academic research by the
## authors and co-workers. If you use it, please support the project by
## citing the appropriate journal articles.
## 
## 
## ====================
## PACKAGE: MASS 
## ====================
## To cite the MASS package in publications use:
## 
##   Venables, W. N. & Ripley, B. D. (2002) Modern Applied Statistics with
##   S. Fourth Edition. Springer, New York. ISBN 0-387-95457-0
## 
## A BibTeX entry for LaTeX users is
## 
##   @Book{,
##     title = {Modern Applied Statistics with S},
##     author = {W. N. Venables and B. D. Ripley},
##     publisher = {Springer},
##     edition = {Fourth},
##     address = {New York},
##     year = {2002},
##     note = {ISBN 0-387-95457-0},
##     url = {https://www.stats.ox.ac.uk/pub/MASS4/},
##   }
## 
## 
## ====================
## PACKAGE: MVN 
## ====================
## To cite MVN in publications use:
## 
##   Korkmaz S, Goksuluk D, Zararsiz G. MVN: An R Package for Assessing
##   Multivariate Normality. The R Journal. 2014; 6(2):151-162.
## 
## A BibTeX entry for LaTeX users is
## 
##   @Article{,
##     title = {MVN: An R Package for Assessing Multivariate Normality.},
##     author = {Selcuk Korkmaz and Dincer Goksuluk and Gokmen Zararsiz},
##     journal = {The R Journal},
##     year = {2014},
##     volume = {6},
##     number = {2},
##     pages = {151--162},
##     url = {https://journal.r-project.org/archive/2014-2/korkmaz-goksuluk-zararsiz.pdf},
##   }
## 
## 
## ====================
## PACKAGE: RVAideMemoire 
## ====================
## To cite package 'RVAideMemoire' in publications use:
## 
##   HERVE M (2023). _RVAideMemoire: Testing and Plotting Procedures for
##   Biostatistics_. R package version 0.9-83-7,
##   <https://CRAN.R-project.org/package=RVAideMemoire>.
## 
## A BibTeX entry for LaTeX users is
## 
##   @Manual{,
##     title = {RVAideMemoire: Testing and Plotting Procedures for Biostatistics},
##     author = {Maxime HERVE},
##     year = {2023},
##     note = {R package version 0.9-83-7},
##     url = {https://CRAN.R-project.org/package=RVAideMemoire},
##   }
## 
## ATTENTION: This citation information has been auto-generated from the
## package DESCRIPTION file and may need manual editing, see
## 'help("citation")'.
## 
## 
## ====================
## PACKAGE: vegan 
## ====================
## To cite package 'vegan' in publications use:
## 
##   Oksanen J, Simpson G, Blanchet F, Kindt R, Legendre P, Minchin P,
##   O'Hara R, Solymos P, Stevens M, Szoecs E, Wagner H, Barbour M,
##   Bedward M, Bolker B, Borcard D, Carvalho G, Chirico M, De Caceres M,
##   Durand S, Evangelista H, FitzJohn R, Friendly M, Furneaux B, Hannigan
##   G, Hill M, Lahti L, McGlinn D, Ouellette M, Ribeiro Cunha E, Smith T,
##   Stier A, Ter Braak C, Weedon J, Borman T (2025). _vegan: Community
##   Ecology Package_. R package version 2.6-10,
##   <https://CRAN.R-project.org/package=vegan>.
## 
## A BibTeX entry for LaTeX users is
## 
##   @Manual{,
##     title = {vegan: Community Ecology Package},
##     author = {Jari Oksanen and Gavin L. Simpson and F. Guillaume Blanchet and Roeland Kindt and Pierre Legendre and Peter R. Minchin and R.B. O'Hara and Peter Solymos and M. Henry H. Stevens and Eduard Szoecs and Helene Wagner and Matt Barbour and Michael Bedward and Ben Bolker and Daniel Borcard and Gustavo Carvalho and Michael Chirico and Miquel {De Caceres} and Sebastien Durand and Heloisa Beatriz Antoniazi Evangelista and Rich FitzJohn and Michael Friendly and Brendan Furneaux and Geoffrey Hannigan and Mark O. Hill and Leo Lahti and Dan McGlinn and Marie-Helene Ouellette and Eduardo {Ribeiro Cunha} and Tyler Smith and Adrian Stier and Cajo J.F. {Ter Braak} and James Weedon and Tuomas Borman},
##     year = {2025},
##     note = {R package version 2.6-10},
##     url = {https://CRAN.R-project.org/package=vegan},
##   }
## 
## 
## ====================
## PACKAGE: NbClust 
## ====================
## To cite NbClust in publications use:
## 
##   Malika Charrad, Nadia Ghazzali, Veronique Boiteau, Azam Niknafs
##   (2014). NbClust: An R Package for Determining the Relevant Number of
##   Clusters in a Data Set. Journal of Statistical Software, 61(6), 1-36.
##   URL http://www.jstatsoft.org/v61/i06/.
## 
## A BibTeX entry for LaTeX users is
## 
##   @Article{,
##     title = {{NbClust}: An {R} Package for Determining the Relevant Number of Clusters in a Data Set},
##     author = {Malika Charrad and Nadia Ghazzali and V{\'e}ronique Boiteau and Azam Niknafs},
##     journal = {Journal of Statistical Software},
##     year = {2014},
##     volume = {61},
##     number = {6},
##     pages = {1--36},
##     url = {https://www.jstatsoft.org/v61/i06/},
##   }
## 
## 
## ====================
## PACKAGE: biotools 
## ====================
## To cite 'biotools' in publications use:
## 
##   da Silva, A. R. (2021) biotools: Tools for Biometry and Applied
##   Statistics in Agricultural Science. R package version 4.2.
##   https://cran.r-project.org/package=biotools
## 
##   da Silva, A.R.; Malafaia, G.; Menezes, I.P.P. (2017) biotools: an R
##   function to predict spatial gene diversity via an individual-based
##   approach. Genetics and Molecular Research, 16. doi:
##   10.4238/gmr16029655
## 
## To see these entries in BibTeX format, use 'print(<citation>,
## bibtex=TRUE)', 'toBibtex(.)', or set
## 'options(citation.bibtex.max=999)'.
## 
## 
## ====================
## PACKAGE: rrcov 
## ====================
## To cite the package 'rrcov' in publications use:
## 
##   Todorov V, Filzmoser P (2009). "An Object-Oriented Framework for
##   Robust Multivariate Analysis." _Journal of Statistical Software_,
##   *32*(3), 1-47. doi:10.18637/jss.v032.i03
##   <https://doi.org/10.18637/jss.v032.i03>.
## 
## A BibTeX entry for LaTeX users is
## 
##   @Article{,
##     title = {An Object-Oriented Framework for Robust Multivariate Analysis},
##     journal = {Journal of Statistical Software},
##     author = {Valentin Todorov and Peter Filzmoser},
##     year = {2009},
##     volume = {32},
##     number = {3},
##     pages = {1--47},
##     doi = {10.18637/jss.v032.i03},
##   }
## 
## 
## ====================
## PACKAGE: DescTools 
## ====================
## To cite package 'DescTools' in publications use:
## 
##   Signorell A (2025). _DescTools: Tools for Descriptive Statistics_. R
##   package version 0.99.59,
##   <https://CRAN.R-project.org/package=DescTools>.
## 
## A BibTeX entry for LaTeX users is
## 
##   @Manual{,
##     title = {DescTools: Tools for Descriptive Statistics},
##     author = {Andri Signorell},
##     year = {2025},
##     note = {R package version 0.99.59},
##     url = {https://CRAN.R-project.org/package=DescTools},
##   }
## 
## 
## ====================
## PACKAGE: compositions 
## ====================
## To cite package 'compositions' in publications use:
## 
##   van den Boogaart KG, Tolosana-Delgado R, Bren M (2024).
##   _compositions: Compositional Data Analysis_. R package version 2.0-8,
##   <https://CRAN.R-project.org/package=compositions>.
## 
## A BibTeX entry for LaTeX users is
## 
##   @Manual{,
##     title = {compositions: Compositional Data Analysis},
##     author = {K. Gerald {van den Boogaart} and Raimon Tolosana-Delgado and Matevz Bren},
##     year = {2024},
##     note = {R package version 2.0-8},
##     url = {https://CRAN.R-project.org/package=compositions},
##   }
## 
## ATTENTION: This citation information has been auto-generated from the
## package DESCRIPTION file and may need manual editing, see
## 'help("citation")'.
## 
## 
## ====================
## PACKAGE: zCompositions 
## ====================
## To cite zCompositions in publications, please use:
## 
##   Palarea-Albaladejo J, Martín-Fernández JA (2015). "zCompositions - R
##   package for multivariate imputation of left-censored data under a
##   compositional approach." _Chemometrics and Intelligent Laboratory
##   Systems_, *143*, 85-96. doi:10.1016/j.chemolab.2015.02.019
##   <https://doi.org/10.1016/j.chemolab.2015.02.019>.
## 
## A BibTeX entry for LaTeX users is
## 
##   @Article{,
##     title = {zCompositions -- R package for multivariate imputation of left-censored data under a compositional approach},
##     author = {J. Palarea-Albaladejo and J. A. Martín-Fernández},
##     journal = {Chemometrics and Intelligent Laboratory Systems},
##     year = {2015},
##     volume = {143},
##     pages = {85--96},
##     doi = {10.1016/j.chemolab.2015.02.019},
##   }
## 
## 
## ====================
## PACKAGE: robCompositions 
## ====================
## To cite package 'robCompositions' in publications you might use:
## 
##   Matthias Templ, Karel Hron, Peter Filzmoser (2011). robCompositions:
##   an R-package for robust statistical analysis of compositional data.
##   In V. Pawlowsky-Glahn and A. Buccianti, editors, Compositional Data
##   Analysis. Theory and Applications, pp. 341-355, John Wiley & Sons,
##   Chichester (UK) .
## 
##   Peter Filzmoser, Karel Hron, Matthias Templ (2018). Applied
##   Compositional Data Analysis. With Worked Examples in R. Springer
##   Series in Statistics. Springer International Publishing, Cham,
##   Switzerland .
## 
## To see these entries in BibTeX format, use 'print(<citation>,
## bibtex=TRUE)', 'toBibtex(.)', or set
## 'options(citation.bibtex.max=999)'.
## 
## 
## ====================
## PACKAGE: gmGeostats 
## ====================
## To cite package 'gmGeostats' in publications use:
## 
##   Tolosana-Delgado R, Mueller U (2023). _gmGeostats: Geostatistics for
##   Compositional Analysis_. R package version 0.11.3,
##   <https://CRAN.R-project.org/package=gmGeostats>.
## 
## A BibTeX entry for LaTeX users is
## 
##   @Manual{,
##     title = {gmGeostats: Geostatistics for Compositional Analysis},
##     author = {Raimon Tolosana-Delgado and Ute Mueller},
##     year = {2023},
##     note = {R package version 0.11.3},
##     url = {https://CRAN.R-project.org/package=gmGeostats},
##   }  
 
 


 

 

 

 

 


 
 

 
 
